# Proteomic Analysis of the Human Skin Proteome after *In Vivo* Treatment with Sodium Dodecyl Sulphate

**DOI:** 10.1371/journal.pone.0097772

**Published:** 2014-05-21

**Authors:** Erika Parkinson, Paul Skipp, Maja Aleksic, Andrew Garrow, Tony Dadd, Michael Hughes, Geraldine Clough, C. David O′Connor

**Affiliations:** 1 Centre for Biological Sciences, University of Southampton, Southampton, United Kingdom; 2 Centre for Proteomic Research, Institute for Life Sciences, University of Southampton, Southampton, United Kingdom; 3 Safety & Environmental Assurance Centre, Unilever, Colworth Science Park, Sharnbrook, United Kingdom; 4 Unilever Clinicals, Unilever Research, Colworth Science Park, Sharnbrook, United Kingdom; 5 Institute for Developmental Sciences, School of Medicine, University of Southampton, Southampton, United Kingdom; National Central University, Taiwan

## Abstract

**Background:**

Skin has a variety of functions that are incompletely understood at the molecular level. As the most accessible tissue in the body it often reveals the first signs of inflammation or infection and also represents a potentially valuable source of biomarkers for several diseases. In this study we surveyed the skin proteome qualitatively using gel electrophoresis, liquid chromatography tandem mass spectrometry (GeLC-MS/MS) and quantitatively using an isobaric tagging strategy (iTRAQ) to characterise the response of human skin following exposure to sodium dodecyl sulphate (SDS).

**Results:**

A total of 653 skin proteins were assigned, 159 of which were identified using GeLC-MS/MS and 616 using iTRAQ, representing the most comprehensive proteomic study in human skin tissue. Statistical analysis of the available iTRAQ data did not reveal any significant differences in the measured skin proteome after 4 hours exposure to the model irritant SDS.

**Conclusions:**

This study represents the first step in defining the critical response to an irritant at the level of the proteome and provides a valuable resource for further studies at the later stages of irritant exposure.

## Introduction

Skin is the largest and most extensive organ in the human body, accounting for some 15% of total body weight and fulfilling a number of diverse functions. Importantly, it represents a major barrier protecting the body from physical, chemical and microbial impacts. With its ability to generate both innate and adaptive immune responses, the skin forms an essential part of the immune system [1]. As a primary defence organ, it is also involved in thermoregulation, transduction of sensory information, protection from UV radiation and dehydration as well as vitamin production [2]–[4].

The functional diversity of skin is reflected in its complex architecture and differentiation. The uppermost layer, the epidermis, is separated by a basal membrane from the inner layer, the dermis. While the dermis is composed of well vascularised dense fibro-elastic connective tissue, the epidermis is an avascular stratified squamous epithelium containing four distinct cell types, principally keratinocytes, but also melanocytes, Langerhan cells and Merkel cells [5]. During terminal differentiation, keratinocytes produce keratin intermediate filaments, which are cross-linked by transglutaminases. Keratinocytes undergo apoptosis resulting in the characteristic cornified epidermis [6]. Skin additionally contains a variety of structures, such as hair follicles, sweat and sebaceous glands, that traverse both layers. During pathological processes major changes in the cellular composition of skin can occur; for example, infiltration by a large number of normally non-resident cells (neutrophils, eosinophils, natural killer cells) during inflammation.

At the molecular level our understanding of the essential physiological functions of skin, and in particular the qualitative and quantitative changes of the proteome induced by exposure to chemicals, is currently incomplete. Pioneering studies by Celis and co-workers have identified a large number of proteins from cultured human and mouse keratinocytes [7]. Additionally, studies in sodium dodecyl sulphate (SDS) induced irritation/inflammation of skin have revealed potential markers and hence provide a good starting point for larger, quantitative studies into this and other skin conditions [8], [9]. Our ultimate goal is to use proteomic approaches to investigate skin responses to chemical exposure. Studying the changes in inflammatory, allergic and other toxicological processes that are triggered in such circumstances may shed light on the molecular processes associated with particular pathologies. We are particularly interested in understanding types and relative levels of nucleophilic amino acids in proteins expressed in skin (‘nucleophilic make-up’), as well as changes induced following topical application of chemicals. Nucleophiles are key targets for covalent modification by reactive chemicals (or reactive metabolites). These conjugation events generate macromolecular immunogens as the small chemicals are invisible to the immune system on their own. It is this event that has long been postulated as causative of skin allergy [10]–[12]. Qualitative and quantitative insights into skin proteome and its ‘nucleophilic make-up’ may lead to a more physiologically relevant interpretation of *in chemico* reactivity data [13].

In this paper we describe a survey of the skin proteome together with an analysis of proteome changes induced by relatively short exposure to the well characterised irritant SDS. Following optimisation of conditions that allowed the rapid solubilisation of skin, proteins from human biopsy samples were analysed by gel electrophoresis, liquid chromatography tandem mass-spectrometry (GeLC-MS/MS) [14], [15] and by the isobaric tagging strategy, iTRAQ [16]. In the former method proteins are separated by conventional 1-D gel electrophoresis prior to *in situ* trypsin treatment. The resulting tryptic peptides are recovered, separated by nano-scale liquid chromatography (nano-LC), and identified by tandem MS and database searching. In the second approach, tryptic peptides are labelled at their primary amines with specific isobaric tags. The labelled peptides are then combined, fractionated, and identified by tandem MS. As the isobaric tags release reporter ions into a region of the mass spectrum that is chemically silent with respect to amino acids and peptides, it is also possible to obtain quantitative information on the peptides and hence the proteins from which they originate. A large number of skin components were uncovered and included keratins, heat shock proteins, proteases, some constituents of adhesion complexes specifically expressed during cornification and proteins involved in the oxidative stress response. We further explore the skin proteome qualitatively and quantitatively to obtain a thorough understanding of the types and relative levels of key nucleophilic amino acids, which could be potential targets for covalent modification (haptenation) by chemical allergens [10]–[12]. To our knowledge, this study represents the most comprehensive quantitative proteomic survey of human skin to date.

## Methods

### Ethics Statement

Ethics was approved for this study (study number: 36-DRM-BPY-06-001) by the Independent Ethics Committee of 4Front Research (from 4th Dec 2006 for 1 year). All participants provided written informed consent and sample collection was conducted according to the principles expressed in the Declaration of Helsinki.

### Sample preparation

#### Sample Collection

Human skin samples (40–52 mg of tissue) from various body sites, used for the method optimisation and for surveying the skin proteome via GeLC-MS/MS, were obtained with informed consent from patients attending dermatology clinics at the Royal South Hants Hospital, UK. Samples were snap frozen in liquid nitrogen within 5 minutes of resection and stored at −80°C prior to analysis.

For the quantitative proteomic analysis, skin biopsy samples were collected by 4-Front Research (Manchester) from healthy volunteers after exposure to the model irritant SDS. Ethical approval was granted for this study and volunteers gave informed written consent. 2–5% SDS (Sigma-Aldrich) in water, or a water-only control, was applied to 18 mm Hill Top chambers, which were applied to the volar forearm of 35 male volunteers for 4 hours. The exact concentrations of SDS that were applied to the forearms of the volunteers can be found in **Table S1 in File S1**. The precise concentration was determined during initial screening and was the minimum that gave an ongoing erythematous response. Erythema reactions were assessed, as described in Basketter *et al.* [32], 1 hour after patch removal prior to 4 mm punch biopsies being taken from the SDS-treated and control (water-treated) sites.

#### Maceration of tissue samples

Biopsy samples were washed twice with 500 µl of 50 mM triethylammonium bicarbonate buffer (TEAB) to remove excess blood and placed into pre-chilled tubes containing ceramic lysing beads (Matrix D – QbioGene, Cambridge, UK) and 400 µl of the appropriate buffer. Five solubilisation buffers were compared, with the following compositions: Buffer 1 (0.5% Nonidet P-40, 0.5% sodium deoxycholate, 10 mM EDTA in 100 mM TEAB); Buffer 2 (7 M Urea, 2 M thiourea, 0.5% Amidosulfobetaine-14 in 100 mM TEAB); Buffer 3 (7 M Urea, 2 M thiourea, 6 M guanidine HCl in 100 mM TEAB); Buffer 4 (0.1% Rapigest in 100 mM TEAB); Buffer 5 (0.1% SDS in 100 mM TEAB). Each tube was then processed using a Savant FastPrep macerator (Thermo Scientific, Loughborough, UK) for 7 cycles of 45 seconds; speed setting 6. The samples were chilled on ice for one minute between cycles and centrifuged for 2 minutes at 9000 x *g* in a microcentrifuge after cycle 3 and for 5 minutes at 9000 x *g* after cycle 7. The tissue lysate was collected and the beads washed by adding 100 µl of the selected buffer to the tube (containing the insoluble material), vortexing for one minute and centrifuging for 2 minutes. The supernatant and previously collected tissue lysate were pooled and stored on ice prior to protein quantitation. The insoluble material remaining after maceration was collected separately.

### Estimation of protein concentration

The protein concentrations of the samples generated after maceration using Buffers 1, 4 and 5 were determined using a bicinchoninic acid (BCA) method [33], [34], using a kit from Sigma-Aldrich (Sigma, Poole, UK). Due to the incompatibility of urea with the BCA assay, samples prepared in Buffers 2 and 3 were determined using the Bradford assay (Bio-Rad, Hemel Hempstead, UK).

### GeLC-MS/MS studies

Following maceration, both soluble (40 µg) and insoluble skin material were denatured in final sample buffer containing 10 mM dithiothreitol at 70°C for 10 minutes. The denatured samples were resolved in a NuPage 4–12% Bis-Tris gel (Invitrogen, Paisley, UK) at 200 V for approximately 45 minutes. Following staining with Colloidal Coomassie Blue, gel tracks (7 cm length) were excised, cut into 25 equal-sized pieces and subjected to *in situ* trypsin digestion using the method of Shevchenko *et al* [35]. The resulting peptides were extracted and separated by nanocapillary liquid chromatography (LC) prior to tandem mass spectrometry.

### iTRAQ labelling

Isobaric tags for relative and absolute quantitation (iTRAQ) produce MS/MS signature ions at *m*/*z* 114.1, 115.1, 116.1, and 117.1 (4-plex) with ions also at *m/z* 113.1, 118.1, 119.1 and 121.1 (8-plex) an area of the mass spectrum which has minimal background noise, whilst the relative areas of these peaks correspond with the proportions of the labeled peptides. This approach allows the relative quantitation of expressed proteins between either a maximum of 4 (4-plex) or 8 samples (8-plex) within a single experiment [16]. iTRAQ labelling of samples was performed according to the manufacturer's instructions (Applied Biosystems, Warrington, UK).

To each of four samples containing ∼150 µg of sample in ≤34 µl of 0.5 M TEAB, 1 µl of 2% SDS and 2 µl of the reducing agent, 50 mM (tris-(2-carboxyethyl) phosphine was added and mixed. Samples were incubated at 60°C for 1 h. Methyl methane-thiosulfonate in isopropanol (1 µl of 200 mM) was then added and incubated for a further 10 min at room temperature. Proteins were digested by adding 10 µl of 1 mg/ml trypsin in 80 mM CaCl_2_. Samples were incubated for 16 h at 37°C.

Labelling of tryptic peptides with iTRAQ tags was achieved by mixing the appropriate iTRAQ labelling reagent with the relevant sample, and incubation at room temperature for 1 h or 2 h for the 4-plex and 8-plex tags, respectively. In the latter case, care was taken to ensure the pH was <7.5 by adding up to 5 µl of TEAB. The main study was performed using the 4-plex iTRAQ tags where the control and test samples from volunteer X were labelled with isobaric tags 114 and 115, respectively, whereas, the control and test samples obtained from volunteer Y were respectively labelled with isobaric tags 116 and 117. The samples were then combined and lyophilized *in vacuo*. As control and test samples were obtained from 35 participants, seventeen 4-plex experiments were prepared with control and test samples from two participants and a final 2-plex experiment contained the control and test sample from the 35^th^ participant. For the reproducibility study, the 8-plex iTRAQ tags were used where the control and test samples from volunteer F37 and F38 were labelled in duplicate.

### Sample pre-processing prior to MS

The combined iTRAQ peptide mixtures were separated by reverse phase chromatography using a Dionex Ultimate LC system and Jupiter C18 reverse phase column (2.1 mm i.d., x 250 mm, 4 µm), (Phenomenex, Macclesfield, UK). Samples were solubilised in 500 µl of Buffer A (5% (v/v) acetonitrile, 0.1% formic acid) and loaded onto the column using a 500 µl loop. The loaded sample was injected and washed with Buffer A for 20 min at 200 µl/min to remove excess reagent. Peptides were eluted with a linear gradient of 0–80% Buffer B (95% acetonitrile, 0.1% formic acid) at 200 µl/min with fractions collected at 1 min intervals. Peptide elution was monitored at 214, 235 and 280 nm. Fractions were lyophilised *in vacuo* prior to MS analysis.

### Mass spectrometry analysis

Peptide samples extracted from 1D-SDS PAGE of soluble and insoluble skin material and iTRAQ labelled samples after the sample pre-processing stage were subjected to MS analysis as follows. 30 µl of 10% acetonitrile containing 0.1% formic acid was added to each sample and loaded onto a reverse phase trap column (5 mm×300 µm, i.d., Dionex, Camberley, UK). The samples were washed for 10 minutes with buffer A prior to the analytical nano-LC separation using a Pepmap C18 Reverse phase column (3 µm, 150 mm×75 µm, i.d., Dionex, Camberley, UK). Fractions were separated over 120 min using a gradient of 0 to 80% Buffer B at 200 nl/min) and electrosprayed into the mass spectrometer. All data were acquired using a Q-tof Global Ultima (Waters Ltd, Manchester, UK) fitted with a nanoLockSpray source. A survey scan was acquired from *m/z* 350 to 1700 with the switching criteria for MS to MS/MS including ion intensity and charge state. The collision energy used to perform MS/MS was varied according to the mass and charge state of the eluting peptide.

The spectra generated in this study has been submitted to the PRIDE database; Project Accession PRD000149 (to view please use reviewer account username: review90658 and password: WTYhPfPG).

### Data analysis

Peak lists were generated using ProteinLynx Global Server 2.2.5 (Waters Ltd, Manchester, UK). The following parameters were used for processing the MS/MS spectra; normal background subtraction with a 25% background threshold and medium de-isotoping with a threshold of 1%; no smoothing was performed.

Peak lists were submitted to the MASCOT search engine version 2.2.1 (MatrixScience, London, UK) and the data searched against a human NCBInr protein sequence database, containing a total of 40877 protein sequences (accessed 10^th^ May 2007). The corresponding quantitative information using the iTRAQ reporter ions, and incorporating isotope correction factors, was also obtained via MASCOT.

Database searches allowed a maximum of one missed cleavage for tryptic digestion while more specifically, for GeLC-MS/MS data the allowed fixed and variable modifications were carboxy-amidomethylation of cysteine and the oxidation of methionine, respectively; for iTRAQ data fixed modifications for methyl methane-thiosulfonation of cysteine and the N-terminus and lysine side chains using either the 4-plex or 8-plex iTRAQ label were allowed (Applied Biosystems, Warrington, UK). Variable modification for the oxidation of methionine and iTRAQ modification of tyrosine were also allowed. Precursor ion and sequence ion mass tolerances were set at 100 ppm and 0.2 Da respectively. Protein identifications required the assignment of ≥2 different peptides with a significance threshold for accepting a match of p<0.05. The protein ratios were calculated using MASCOT version 2.2.1, where the peptide ratios were weighted and median normalisation was performed, automatic outlier removal was chosen and the peptide threshold was set to ‘at least homology’. False discovery rates were calculated by running all spectra against a decoy database using the MASCOT software, the false discovery rate was invariably <2.1%.

Gene Identifiers for the proteins, obtained after MASCOT searching, were cross-referenced with UniProt identifiers using the open-access Protein Identifier Cross Reference tool (http://www.ebi.ac.uk/Tools/picr/), with searches limited to human proteins and mapped to the Swiss-Prot and TrEMBL databases. Proteins were further annotated with theoretical mass and p*I* information (http://www.expasy.ch/tools/pi_tool.html), where Uniprot identifiers were not available, mass and p*I* were taken from the MASCOT search data files.

#### Data integration, processing and statistical analyses

Prior to formal statistical analysis, raw intensity values were corrected for minor cross-channel isotope bias using formulae supplied by Applied Biosystems, average intensities calculated for each peptide across multiple queries, and proteins removed that were identified by only one peptide. Data from multiple iTRAQ experiments were then analysed using SAS v9.2 (SAS Institute Inc., Cary, NC, USA) using the modelling approach of Hill *et al*. [36]. Intensities were transformed using log base 2 and normalised using backfitting as suggested by Oberg *et al*. [37]. This was implemented using an iterative fit to experimental and protein/peptide imbalances with code originally authored by Douglas W. Mahoney at the Mayo Foundations for Medical Education and Research (see Supporting Information for the Oberg paper).

Proteins identified in 5 or fewer individuals were removed from the analysis, leaving 136 proteins. A general linear mixed model was then applied to the normalised log-transformed intensities for each protein in turn, with the two-level factor treatment/control as the only fixed explanatory effect, and a single random effect (individual*treatment) to model the within-individual correlations of the peptides. The addition of the random effect in this analysis also ensures that an appropriate number of degrees of freedom is used in the statistical test for treatment vs. control and therefore avoids “spurious accuracy” in the results. Estimates and 95% C.I.s for the difference between treatment and control were produced from the model and back-transformed from the log-scale to a ratio (treatment/control) scale. P-values were output and a Bonferroni correction applied.

#### Gene ontology analysis

Uniprot identifiers from the GeLC-MS/MS and iTRAQ experiments were submitted into Gene Ontology (GO) Term Mapper (http://go.princeton.edu/cgi-bin/GOTermMapper) and were searched against the goa_human (GOA GO slim) and the goa_human (Generic GO slim) terms for cellular compartment and biological process. The most relevant terms were taken from the GOA and Generic GO slim searches and combined into one file. The numbers of proteins annotated to each term are expressed as a percentage of the total number of Uniprot identifiers submitted into the GO Term Mapper.

#### Estimation of nucleophilic abundance within the identified skin proteome

Quantification of nucleophilic abundance was performed according to the calculations described by Ishihama et al [38]. This work applies the emPAI (Exponentially Modified Protein Abundance Index) value for a population of proteins identified through mass spectrometry to estimate their absolute abundance within a sample. The emPAI value is a derivation of the total number of observed peptides per protein divided by the total number of observable peptides per protein. The emPAI values used here were calculated in Mascot (ver 2.2) per experiment, representing two patients, and converted to protein weight fraction, as described in equation 1 [38]. Based on measurements from section 2.2 (∼50 µg protein per mg skin), the weight fraction was converted to absolute weight per mg skin tissue (equation 2). This was then converted to moles per mg tissue (equation 3) and copy number of each protein (equation 4).

For each protein, FASTA sequences were downloaded from UniProt (where available) and NCBI Protein, via their respective webservices [39], [40]. A computational approach was used to count the nucleophilic residues, Arg, Cys, His, Lys and Tyr within each sequence. Nucleophile counts were multiplied by copy number, summed and proportions of each were compared against the non-nucleophilic residues. Additionally, where information on disulphide-bridged Cys residues was available from UniProt, this was subtracted from the sum of available nucleophiles. Results for Haemoglobin and Serum Albumin proteins were removed from the final analysis as these were believed to be present as a result of the skin biopsy procedure.

Equation 1: Protein content (weight fraction), P_1_ = 
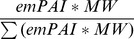


Equation 2: µg protein per mg tissue, P_2_ = P_1_×50* (*∼50 µg protein per mg skin)

Equation 3: Moles protein per mg tissue, P_3_ = 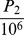
×MW

Equation 4: Number of proteins per mg tissue  = P3×A (mean taken across 18 iTRAQ experiments with emPAI values)

Where MW  =  molecular weight and A = Avogadro's constant, 6.0221415×10^23^

## Results

### An optimised, iTRAQ-compatible method for solubilising skin proteins

It was first necessary to devise an efficient procedure for the solubilisation of skin components that would be compatible with quantitative proteomic procedures such as iTRAQ. Proteins were extracted from human skin biopsy samples by maceration using a ceramic bead beating system and the degree of protein solubilisation was optimised using buffers containing different combinations of detergents and chaotropic agents. The amount of protein extracted per milligram of tissue was similar for all five buffers used, although Buffers 4 and 5 gave the least and most solubilised protein, respectively (**Table S2 in File S1**). Buffers 2 and 3 solubilised proteins with a wide range of molecular weights but left a significant number of proteins behind in the insoluble material. Buffers 4 and 5 solubilised a range of proteins and produced a good range of bands in both the high and low molecular weight regions of 1-D gels (**Figure S1 in File S2)**. However, the intensities of individual bands were greater with Buffer 5.

To evaluate the compatibility of the solubilisation buffers with quantitative proteomic strategies such as iTRAQ, equal amounts of solubilised skin proteins prepared in the Buffers 2, 3, 4 and 5 were labelled with iTRAQ tags 114, 115, 116 and 117, respectively, under standard conditions. (Samples solubilised with Buffer 1 were not studied further as it was likely that the NP-40 detergent contained in the buffer would interfere with the processing for MS analysis). Labelled samples were combined and analysed using data-dependent LC-MS/MS. Inspection of the fragmentation spectra indicated that the ion counts for the 117 isobaric tag were generally higher than the average ion counts for the other tags (data not shown). Thus, the data suggest that Buffer 5 (0.1% SDS in 100 mM TEAB) provided the best quality mass spectra and was optimal for solubilisation of proteins from skin biopsy samples. Accordingly, this buffer was used for subsequent GeLC-MS/MS and iTRAQ studies.

### Qualitative survey of the human skin proteome

**Figure 1:**
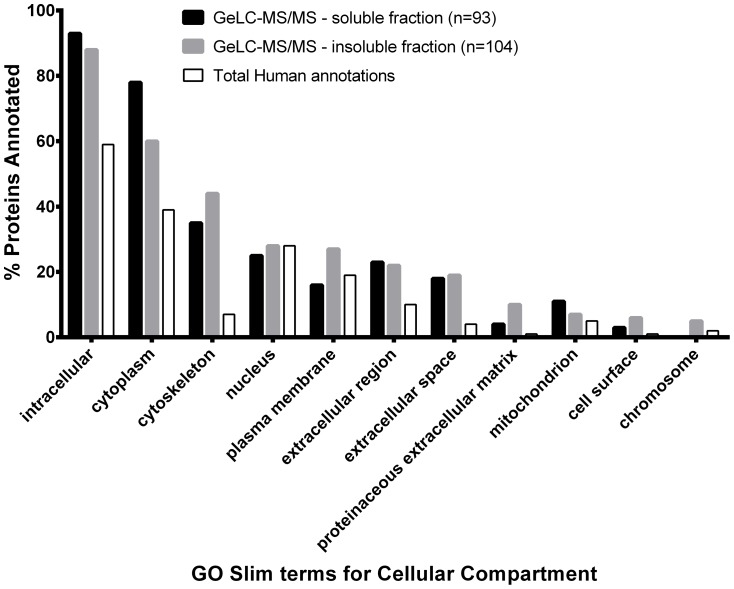
Cellular location of proteins identified from GeLC-MS/MS analysis of human skin. Locations of proteins identified in the GeLC-MS/MS analysis of the soluble and insoluble fractions annotated to the GOA human GO slim terms and the Generic human GO slim terms for cellular compartment, using GO Term Mapper (http://go.princeton.edu/cgi-bin/GOTermMapper). For comparison, the percentage proteins annotated to these terms in the annotated human proteome is also shown.

In view of the paucity of information on the global protein composition of skin, a shotgun proteomics study was carried out to survey the human skin proteome. Conventional SDS-PAGE in conjunction with nanocapillary LC and MS/MS (GeLC-MS/MS) was used to increase the peptide peak and load capacity, thereby increasing the number of components that could be identified. Both the soluble fraction and insoluble fractions obtained from the processing of skin in 0.1% SDS+100 mM TEAB were subjected to GeLC-MS/MS. A comparison of the proteins identified in both fractions in relation to the Gene Ontology terms for cellular compartment revealed that a wide range of proteins were solubilised, including cytoplasmic, cytoskeletal, membrane and extracellular proteins (**Fig 1**). The GeLC-MS/MS dataset was combined with qualitative data from 18 iTRAQ experiments, described in the next section, to maximise coverage of the skin proteome. In all, GeLC-MS/MS and iTRAQ approaches identified 159 and 616 proteins, respectively, of which 122 were common to both. Of the 159 proteins identified in the GeLC-MS/MS studies, 64 and 51 were found exclusively in the insoluble and soluble fractions, respectively. Collectively, the proteins that were revealed had diverse physico-chemical properties suggesting that the sampling strategy was relatively unbiased. For example, the molecular masses of the identified proteins ranged from 11–165 kDa in the soluble fraction and from 10–513 kDa in the insoluble residue; their predicted isoelectric points (p*I*s) ranged from 4.1 to 10.1 in the soluble fraction and from 4.7 to 11.4 in the insoluble residue. Thus, the method developed for the preparation of skin biopsy samples yielded an acceptable range of proteins that were likely to be representative of the expressed skin proteome.

**Figure 2:**
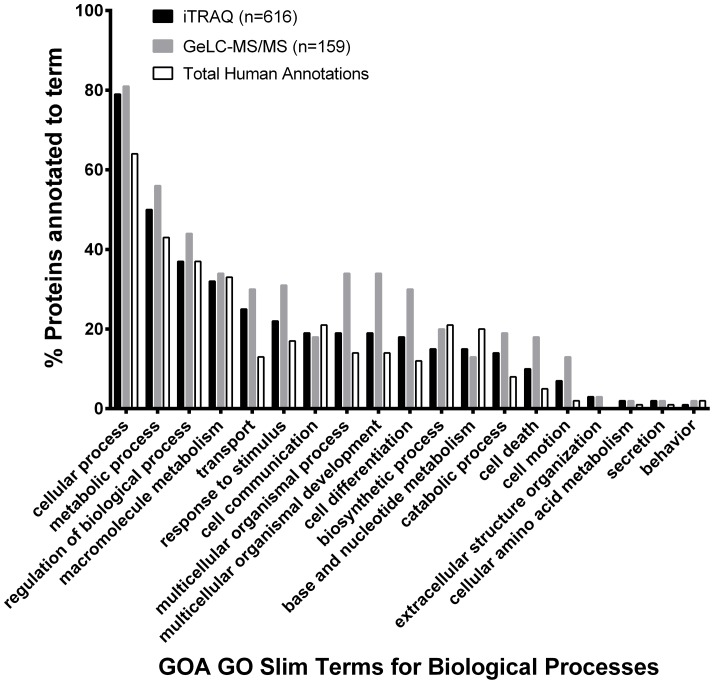
Biological function of all proteins identified after mass spectrometry analysis of human skin. Biological processes associated with proteins identified in human skin using GeLC-MS/MS and iTRAQ analyses. Proteins were annotated with the GOA human GO slim terms for biological process, using GO Term Mapper (http://go.princeton.edu/cgi-bin/GOTermMapper). For comparison, the percentage proteins annotated to these terms in the annotated human proteome is also shown.

A list of the proteins identified in both the GeLC-MS/MS and iTRAQ studies is shown in **Table S3 in File S1**. In keeping with their role in maintaining tissue integrity, a variety of keratins were uncovered, including keratins 1–10, 12–20 and 24–25. All of the keratins were identified in the iTRAQ studies, although many were also associated with the insoluble fraction from the GeLC-MS/MS analysis. Several proteases were identified, including caspase-14 (expression of which was validated by western blot analysis, see **Figure S2 in File S2**), which is involved in filaggrin processing [17], [18], cathepsins D and G, chymase I, carboxypeptidase A3 and tryptase. A number of heat shock proteins (HSPs) were identified, including HSP27, the expression of which has previously been found altered upon SDS exposure in human keratinocytes and excised skin [19], [20]. Constituents of the adhesion complexes specifically expressed during cornification (e.g. periplakin, plakophilin, desmoglein and desmocollin) were observed as well as proteins involved in anti-oxidant defense (e.g. catalase, superoxide dismutase, peroxiredoxin and thioredoxin) [6], [21]. Our analyses showed that skin contains several members of the 14-3-3 family of proteins (e.g. YWHAB, YWHAE, YWHAG, YWHAH, YWHAQ and YWHAZ), which mediate signal transduction by binding to phosphoserine-containing proteins. The molecular rationale for the diversity of such proteins in skin awaits further investigation. Gene Ontology annotations of the proteins identified in the GeLC-MS/MS and iTRAQ experiments for terms associated with biological processes revealed that collectively there is no bias in the types of proteins identified using either GeLC-MS/MS or iTRAQ approaches. The skin proteome appears to contain components involved in a number of different biological processes, including regulatory processes, transport and metabolism (**Fig 2**).

**Figure 3:**
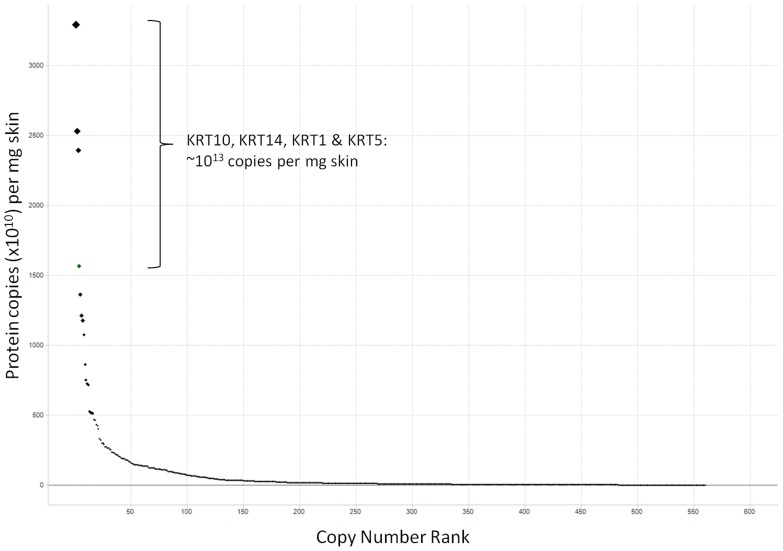
Plot of protein abundance versus abundance ranking. Estimated copy numbers for identified proteins. The steep gradient gives confidence that the most significant members of the skin protein population have been identified. The most prevalent proteins were shown to be in the Keratin family, as expected.

**Figure 4:**
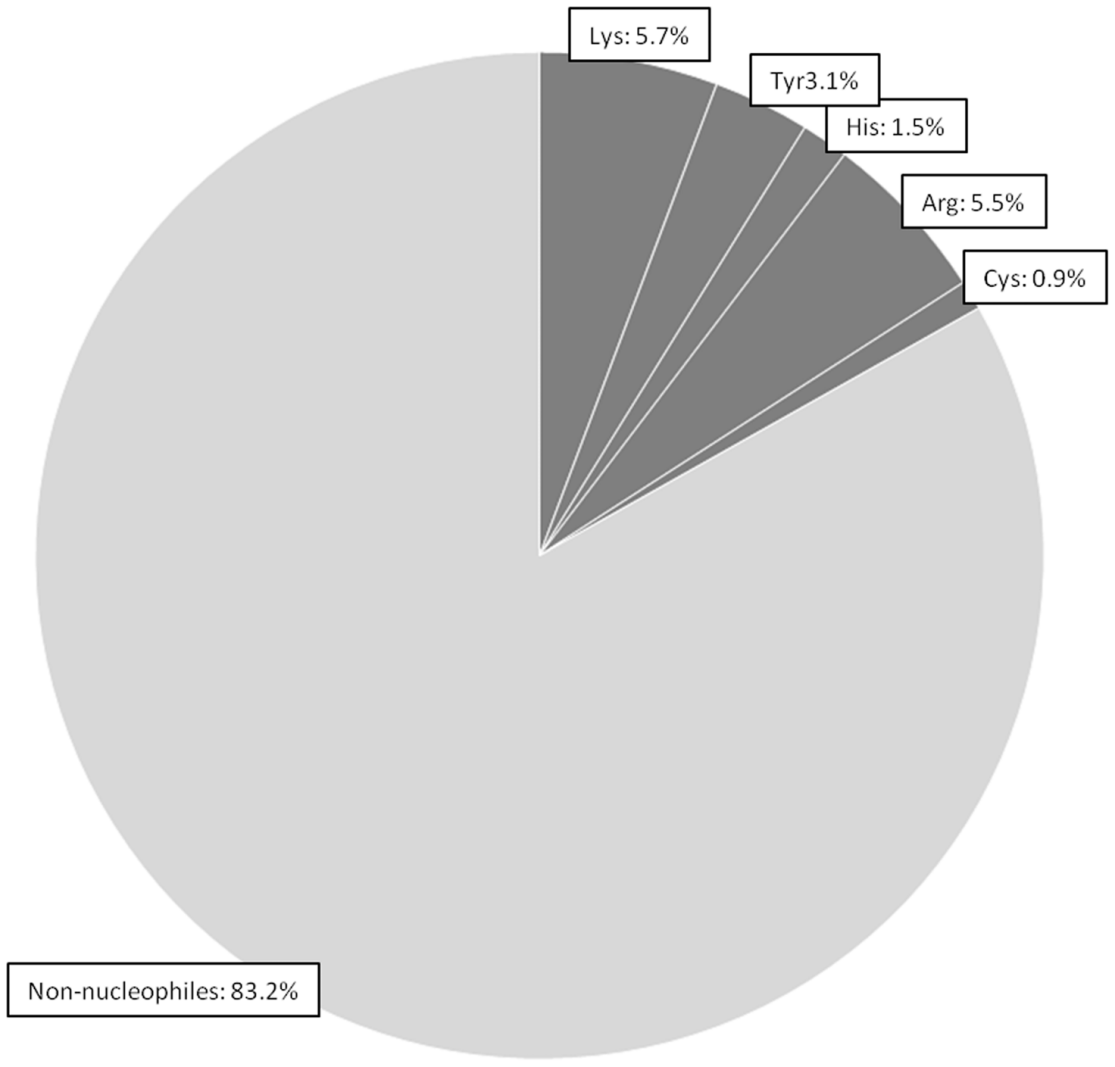
Estimated ratio of non-nucleophiles to nucleophiles with the identified skin proteins. Sequences for proteins identified from the skin biopsy samples were scanned for nucleophilic residues, Cys, His, Lys, Tyr & Arg, and counts were adjusted for protein abundance, which was calculated using emPAI values [38]. Results showed the ratio of non-nucleophiles to nucleophiles to be ∼83:17

The most abundant proteins observed in our samples were members of the keratin family, with an estimate in the region of 10^13^ molecules per mg skin. Haemoglobins and Serum Albumin were present at orders of magnitude higher than the keratins however these were removed from the final calculation as they were believed to be due to blood contamination of the skin samples from the biopsy process. The proteins that could not be confidently identified by mass spectrometry in these samples could potentially affect the calculations of nucleophilic abundance. However, plotting identified protein abundance versus abundance ranking gives reassurance that a representative population of proteins have been accounted for (**Fig 3**). The estimated ratio of non-nucleophiles to nucleophiles in the skin was found to be in the region of 83:17 (**Fig 4**). The proportions of nucleophiles break down into Lys 5.7%, Arg 5.5%, Tyr 3.1%, His 1.5% and Cys not involved in disulphide bridges 0.9%. Total nucleophile proportions were similar in subcellular locations: extracellular 17.8%; membrane, 17.1%: cytoplasm, 17.5%; nucleus, 17.9%.

Results compare well with Cedano *et al* [22], in which 1200 sequences from different subcellular locations were surveyed for residue content. These results follow a similar trend before adjustment for protein abundance and disulphide-bridging. We also surveyed the nucleophilic content from other tissue types as annotated in UniProt. These studies suggest that abundance of key nucleophilic amino acids in skin is comparable to the human proteome overall.

### iTRAQ analysis

To complement the shotgun proteomic studies, an iTRAQ analysis was undertaken to compare alterations in protein expression 4 hours after SDS treatment. The spectra generated in this study were been submitted to the PRIDE database; Project Accession PRD000149.

**Figure 5:**
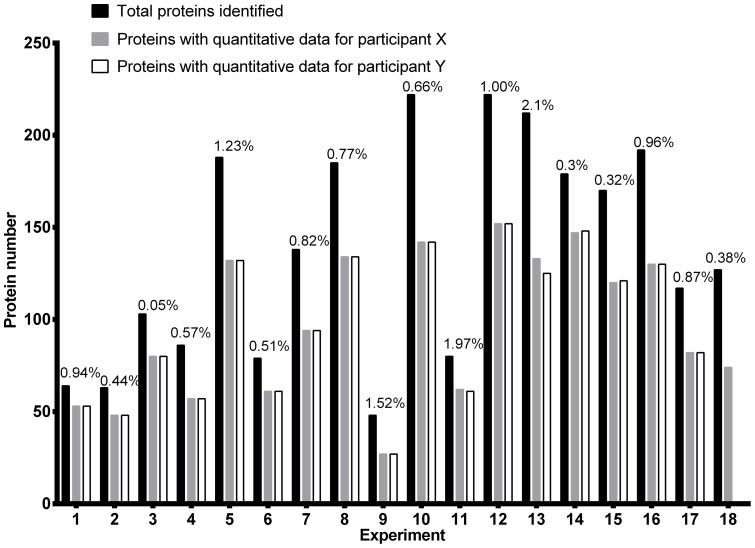
Summary of all proteins, and false discovery rates, identified within each iTRAQ 4-plex experiment. The number of proteins identified in skin samples from different volunteers following MASCOT searches of the NCBI non-redundant human database, including the number of proteins that also had quantitative data associated with each participant. The graph also indicates the number of proteins that yielded quantitative data and the false discovery rates for each experiment, as determined by searching the reverse NCBI non-redundant human database.

The total number of proteins identified in each of the 18 experiments, representing 35 individuals, varied from 48 to 222, with false discovery rates of ≤2.1% (**Fig 5**). By combining the search data from all 18 experiments, a total of 405,054 mass spectra led to the identification of 24,581 peptides, which were assigned to 616 proteins; quantitative data in at least one experiment was obtained for 367 of these proteins. Reproducibility, as assessed by separate analysis of the same samples using 8-plex iTRAQ gave very similar ratio values for the majority of those proteins identified in both the original 4-plex iTRAQ and the 8-plex iTRAQ experiments (**Figure S3 in File S2**). A list of identified proteins, together with their calculated expression ratios in SDS-treated and untreated skin, is shown in **File S3**.

As expected, only modest changes in protein expression were observed following 4 h exposure of skin to SDS, with coefficients of variation for the protein ratios ranging from 0.3%–234% and an average value of 23.5%. However, robust statistical analysis using ANOVA revealed that these changes were not significant, although several proteins showed trends toward up-regulation.

## Discussion

Stratified epithelia cover the entire external and internal surfaces of the human body and serve to prevent water loss as well as to protect against chemical damage, environmental insults and infection. Despite these crucial roles, relatively little is known about the global protein composition of skin and how it changes in response to exposure to noxious substances. In this paper, we have reported an efficient method for solubilisation of skin proteins and its use to conduct a detailed survey of the skin proteome and to study early responses to the irritant SDS.

The new method identified a diversity of proteins, with a broad range of molecular weights and isoelectric points (p*I*s), from many sub-cellular locations (cytoskeletal-, extracellular-, nuclear-, and membrane-associated etc.). These observations suggest that the skin proteome is sampled in a relatively unbiased manner. Furthermore, the relative speed of the new procedure, coupled with its ability to process multiple samples in parallel, allows rigorous and statistically robust clinical proteomic studies on skin.

Previously, proteomic analysis of corneocytes obtained by varnish stripping normal skin identified 77 proteins [23]. In two separate studies, Broccardo *et al* performed LC-MS/MS analysis of lysates of skin cells sampled by tape stripping acute and chronic lesional and non-lesional areas in patients with atopic dermatitis as well as non-atopic individuals and identified 104 and 153 proteins [24], [25]. Using the present method, a survey of human skin proteome, using both GeLC-MS/MS and iTRAQ approaches, we identified 653 proteins, with 39, 69 and 100 proteins overlapping with the studies described above, respectively. While the total repertoire of skin proteins is undoubtedly larger, the identified peptides and proteins provide a framework for further targeted studies. For example, it should now be possible to measure in parallel the levels of all 653 proteins under different cellular conditions using multiple (or selected) reaction monitoring [26]. Further, an extension of this approach can be used to quantify reversible post-translation modifications of these proteins, e.g. phosphorylation, as well as irreversible modifications such as haptenation. The data can also be used to aid deeper mining of the skin proteome. For example, knowledge of the relative abundance of the proteins (as indicated by the number of times that their peptides were sampled during the study), allows the design of better strategies for the depletion of common proteins, thereby allowing low abundance proteins to be uncovered.

In this study we also investigated changes in the skin proteome during the early irritant responses of skin to SDS using an iTRAQ strategy. Relatively few differentially expressed proteins were uncovered as the skin biopsies were taken 1 hour after 4 hours of treatment with SDS, however analysis by ANOVA revealed that these changes were not statistically significant. The co-efficient of variation of the protein ratios ranged from 0.3%–234.4% resulting in a relatively low magnitude of the fold-changes.

This dataset is additionally providing a valuable insight into levels of key protein nucleophilic residues that are potential conjugation targets of electrophilic chemicals or metabolites, the products of which are causative of a variety of adverse effects, most notably delayed type hypersensitivity reactions [10]–[12]. However, current understanding of chemical conjugation to proteins dictates that actual covalent modifications occur only on a small proportion of total protein nucelophiles. Information obtained from studies using single protein models [27]–[31] showed that chemical modification of proteins only occurs in the specific microenvironments within the 3D protein structures which are especially conducive to reactivity, via the influence of the neighbouring side chains on the pKa of the target nucleophiles. Thus more effort is required to account for the role of the 3D protein structure in determining the ultimate availability of nucleophilic amino acid side chains for reaction with exogenous chemical in physiologically relevant conditions. It is important to note that this dataset does not include non-protein nucleophiles that are normally present in all tissues including skin (e.g. glutathione (GSH)), which will contribute to the overall conjugation reactions of the exogenous chemical but will not generate immunogenic species. Understanding not only the nucleophilic make-up of skin tissue but also the effective availability of nucleophiles for conjugation with chemicals will be instrumental in understanding the formation of complete antigens in human skin and their inherent immunogenicity in delayed type hypersensitivity. This information may also help interpretation of chemical reactivity data obtained using simple models of protein nucleophiles in a physiological context [13].

## Conclusions

We have produced the most comprehensive qualitative and quantitative dataset of skin proteome to date and characterised early responses to the irritant SDS at the level of the proteome. Additionally, we used this dataset to investigate types and relative levels of key nucleophilic amino acids, potential targets for haptenation by chemicals. These studies should provide a framework for future investigations to further define critical properties of skin, and in particular its responses to noxious agents.

## Supporting Information

File S1 **Tables S1, S2 and S3. Table S1** The concentrations of SDS that were applied to the forearms of each volunteer in the main study based on the SDS concentrations that elicited a grade 2/3 erythema (using the Human Studies Grading Scheme) during screening. **Table S2** Yields of protein from skin biopsies with different extraction buffers **Table S3** Proteins identified following GeLC-MS/MS of soluble and insoluble fractions, and iTRAQ analysis of skin samples.
(DOC)

File S2 **Figures S1, S2 and S3. Figure S1** SDS PAGE of skin tissue lysates prepared in different buffers. **Figure S2** Western Blot analysis of control and test biopsy samples from 2 volunteers showing expression of caspase-14. **Figure S3** Reproducibility of data in a representative experiment (F37F38) using 4- and 8-plex iTRAQ analysis.
(DOCX)

File S3 **iTRAQ data.** This file contains the expression ratios of control versus SDS-treated for all of the proteins identified within each iTRAQ experiment, where data was available. P-values and co-efficient of variation of the expression ratios across all 18 iTRAQ experiments are also shown.
(XLS)
