# Supplementary material for: Proteomic Analysis of the Human Skin Proteome after In Vivo Treatment with Sodium Dodecyl Sulphate
Source: PLoS One. 2014 May 21;9(5):e97772. doi: 10.1371/journal.pone.0097772 (PMC4029809; doi:10.1371/journal.pone.0097772)
Supplement: File S1 — Tables S1, S2 and S3. Table S1 The concentrations of SDS that were applied to the forearms of each volunteer in the main study based on the SDS concentrations that elicited a grade 2/3 erythema (using the Human Studies Grading Scheme) during screening. Table S2 Yields of protein from skin biopsies with different extraction buffers Table S3 Proteins identified following GeLC-MS/MS of soluble and insoluble fractions, and iTRAQ analysis of skin samples. (DOC) [file pone.0097772.s001.doc]

# File S1

# Table S1 – The concentrations of SDS that were applied to the forearms of each volunteer in the main study based on the SDS concentrations that elicited a grade 2/3 erythema (using the Human Studies Grading Scheme) during screening.

| Volunteer | % SDS | Volunteer | % SDS | Volunteer | % SDS | Volunteer | % SDS |
| --- | --- | --- | --- | --- | --- | --- | --- |
| **F01** | **4** | **F11** | **3** | **F23** | **2** | **F32** | **2** |
| **F02** | **5** | **F15** | **3** | **F24** | **3** | **F35** | **2** |
| **F03** | **3** | **F16** | **2** | **F25** | **3** | **F36** | **5** |
| **F04** | **5** | **F17** | **5** | **F26** | **3** | **F37** | **2** |
| **F05** | **3** | **F18** | **2** | **F27** | **4** | **F38** | **5** |
| **F06** | **4** | **F19** | **5** | **F28** | **4** | **F39** | **5** |
| **F07** | **5** | **F20** | **2** | **F29** | **2** | **F52** | **2** |
| **F08** | **4** | **F21** | **4** | **F30** | **3** | **F56** | **5** |
| **F09** | **2** | **F22** | **2** | **F31** | **3** |  |  |

# Table S2 - Yields of protein from skin biopsies with different extraction buffers

| **Buffer** | **Composition (all in 100 mM TEAB)** | **Mass of tissue (mg)** | **Protein conc. (mg/ml)** | **Total protein (µg)** | **Total protein /mg tissue** |
| --- | --- | --- | --- | --- | --- |
| 1 | 0.5% NP-40, 0.5% sodium deoxycholate, 10 mM EDTA | 45.5 | 6.90 | 2525 | 55.5 µg |
| 2 | 7M Urea, 2 M thiourea, 0.5% ASB-14 | 59.1 | 8.13 | 2764 | 46.8 µg |
| 3 | 7M Urea, 2M thiourea, 6 M guanidine HCl | 44.3 | 6.24 | 2203 | 49.7 µg |
| 4 | 0.1% Rapigest | 51.2 | 6.89 | 2081 | 40.6 µg |
| 5 | 0.1% SDS | 42.2 | 6.32 | 2389 | 56.6 µg |
|  |  |  |  |  |  |

**Table 3** - **Proteins identified following GeLC-MS/MS of soluble and insoluble fractions, and iTRAQ analysis of skin samples**

| **Gi number** | **Protein name** | **Gene** | **Uniprot Identifier** | | **mass** | **p*I*** | **GeLC-MS/MS IS** | **GeLC-MS/MS S** | **iTRAQ** |
| --- | --- | --- | --- | --- | --- | --- | --- | --- | --- |
| gi|4503301 | 2,4-dienoyl CoA reductase 1 precursor | DECR1 | [Q16698](http://www.uniprot.org/uniprot/Q16698) | | 32130 | 8.79 |  |  | ✓ |
| gi|54020720 | 3-hydroxy-3-methylglutaryl-Coenzyme A synthase 1 (soluble) | HMGCS1 | [Q01581](http://www.uniprot.org/uniprot/Q01581) | | 57257 | 5.22 |  | ✓ |  |
| gi|37594469 | 3-hydroxyisobutyryl-Coenzyme A hydrolase isoform 2 | HIBCH | [Q6NVY1](http://www.uniprot.org/uniprot/Q6NVY1) | | 39459 | 6.3 |  |  | ✓ |
| gi|41201737 | 40S ribosomal protein SA (p40) | RPSA | [P08865](http://www.uniprot.org/uniprot/P08865) | | 32702 | 4.79 |  | ✓ |  |
| gi|31377800 | 60kD Ro/SSA autoantigen | TROVE2 | [P10155](http://www.uniprot.org/uniprot/P10155) | | 60631 | 8.27 |  |  | ✓ |
| gi|14249382 | abhydrolase domain containing 14B | ABHD14B | [Q96IU4](http://www.uniprot.org/uniprot/Q96IU4) | | 22332 | 5.94 |  |  | ✓ |
| gi|33667044 | ABI gene family, member 3 (NESH) binding protein | ABI3BP | [Q7Z7G0](http://www.uniprot.org/uniprot/Q7Z7G0) | | 115791 | 9.49 |  |  | ✓ |
| gi|5174389 | acetyl-Coenzyme A acetyltransferase 2 | ACAT2 | [Q9BWD1](http://www.uniprot.org/uniprot/Q9BWD1) | | 41324 | 6.47 |  | ✓ | ✓ |
| gi|5174429 | acetyl-coenzyme A acyltransferase 2 | ACAA2 | [P42765](http://www.uniprot.org/uniprot/P42765) | | 41898 | 8.32 |  | ✓ | ✓ |
| gi|4501867 | aconitase 2 precursor | ACO2 | [Q99798](http://www.uniprot.org/uniprot/Q99798) | | 82374 | 6.85 |  |  | ✓ |
| gi|63055057 | actin beta-like 2 | ACTBL2 | [Q562R1](http://www.uniprot.org/uniprot/Q562R1) | | 41976 | 5.39 |  |  | ✓ |
| gi|5031599 | actin related protein 2/3 complex subunit 2 | ARPC2 | [O15144](http://www.uniprot.org/uniprot/O15144) | | 34311 | 6.84 |  |  | ✓ |
| gi|5031597 | actin related protein 2/3 complex subunit 3 | ARPC3 | [O15145](http://www.uniprot.org/uniprot/O15145) | | 20402 | 8.82 |  |  | ✓ |
| gi|63055057 | Actin, cytoplasmic 1 (Beta-actin). | ACTB | [P60709](http://www.uniprot.org/uniprot/P60709) | | 41710 | 5.29 | ✓ | ✓ |  |
| gi|4501887 | actin, gamma 1 propeptide | ACTG1 | [P63261](http://www.uniprot.org/uniprot/P63261) | | 41766 | 5.31 |  | ✓ |  |
| gi|4501891 | actinin, alpha 1 | ACTN1 | [P12814](http://www.uniprot.org/uniprot/P12814) | | 102993 | 5.25 | ✓ |  | ✓ |
| gi|4501893 | actinin, alpha 2 | ACTN2 | [P35609](http://www.uniprot.org/uniprot/P35609) | | 103788 | 5.31 |  |  | ✓ |
| gi|12025678 | actinin, alpha 4 | ACTN4 | [O43707](http://www.uniprot.org/uniprot/O43707) | | 104788 | 5.27 |  |  | ✓ |
| gi|62990121 | actin-like protein | ACTBL3 | [Q9BYX7](http://www.uniprot.org/uniprot/Q9BYX7) | | 41989 | 5.91 |  |  | ✓ |
| gi|5031571 | actin-related protein 2 isoform b | ACTR2 | [P61160](http://www.uniprot.org/uniprot/P61160) | | 44732 | 6.29 |  |  | ✓ |
| gi|40807491 | acyl-CoA synthetase long-chain family member 1 | ACSL1 | [P33121](http://www.uniprot.org/uniprot/P33121) | | 77893 | 6.81 | ✓ |  | ✓ |
| gi|4557233 | acyl-Coenzyme A dehydrogenase, C-2 to C-3 short chain precursor | ACADS | [P16219](http://www.uniprot.org/uniprot/P16219) | | 41694 | 6.15 |  |  | ✓ |
| gi|4557235 | acyl-Coenzyme A dehydrogenase, very long chain isoform 1 precursor | ACADVL | [P49748](http://www.uniprot.org/uniprot/P49748) | | 66133 | 7.74 |  |  | ✓ |
| gi|22027651 | adaptor-related protein complex 1 beta 1 subunit isoform a | AP1B1 | [Q10567](http://www.uniprot.org/uniprot/Q10567) | | 104540 | 4.94 |  |  | ✓ |
| gi|40018648 | adaptor-related protein complex 3, delta 1 subunit | AP3D1 | [O14617](http://www.uniprot.org/uniprot/O14617) | | 130076 | 8.69 |  |  | ✓ |
| gi|4502171 | adenine phosphoribosyltransferase isoform a | APRT | [P07741](http://www.uniprot.org/uniprot/P07741) | | 19464 | 5.79 |  |  | ✓ |
| gi|4502011 | adenylate kinase 1 | AK1 | [P00568](http://www.uniprot.org/uniprot/P00568) | | 21621 | 8.73 |  |  | ✓ |
| gi|4502013 | adenylate kinase 2 isoform a | AK2 | [P54819](http://www.uniprot.org/uniprot/P54819) | | 26330 | 7.85 |  |  | ✓ |
| gi|5453595 | adenylyl cyclase-associated protein | CAP1 | [Q01518](http://www.uniprot.org/uniprot/Q01518) | | 51692 | 8.29 |  |  | ✓ |
| gi|4502201 | ADP-ribosylation factor 1 | ARF1 | [P84077](http://www.uniprot.org/uniprot/P84077) | | 20553 | 6.36 |  |  | ✓ |
| gi|4502205 | ADP-ribosylation factor 4 | ARF4 | [P18085](http://www.uniprot.org/uniprot/P18085) | | 20367 | 6.81 |  |  | ✓ |
| gi|4502209 | ADP-ribosylation factor 5 | ARF5 | [P84085](http://www.uniprot.org/uniprot/P84085) | | 20386 | 6.35 |  |  | ✓ |
| gi|5453704 | ADP-ribosylation-like factor 6 interacting protein 5 | ARL6IP5 | [O75915](http://www.uniprot.org/uniprot/O75915) | | 21600 | 9.77 |  |  | ✓ |
| gi|61743954 | AHNAK nucleoprotein isoform 1 | AHNAK | [Q09666](http://www.uniprot.org/uniprot/Q09666) | | 628699 | 5.8 |  |  | ✓ |
| gi|4502027 | albumin precursor | ALB | [P02768](http://www.uniprot.org/uniprot/P02768) | | 66429 | 5.67 | ✓ | ✓ | ✓ |
| gi|34577061 | alcohol dehydrogenase 1B (class I), beta polypeptide | ADH1B | [P00325](http://www.uniprot.org/uniprot/P00325) | | 39698 | 8.63 |  |  | ✓ |
| gi|21361176 | aldehyde dehydrogenase 1A1 | ALDH1A1 | [P00352](http://www.uniprot.org/uniprot/P00352) | | 54696 | 6.29 |  |  | ✓ |
| gi|25777739 | aldehyde dehydrogenase 9A1 | ALDH9A1 | [P49189](http://www.uniprot.org/uniprot/P49189) | | 53636 | 5.69 |  | ✓ |  |
| gi|4557305 | aldolase A | ALDOA | [P04075](http://www.uniprot.org/uniprot/P04075) | | 39264 | 8.39 |  | ✓ | ✓ |
| gi|4501881 | alpha 1 actin precursor | ACTA1 | [P68133](http://www.uniprot.org/uniprot/P68133) | | 41790 | 5.23 | ✓ | ✓ | ✓ |
| gi|4502945 | alpha 1 type I collagen preproprotein | COL1A1 | [P02452](http://www.uniprot.org/uniprot/P02452) | | 138911 | 5.66 | ✓ | ✓ | ✓ |
| gi|13435125 | alpha 1 type II collagen isoform 1 | COL2A1 | [P02458](http://www.uniprot.org/uniprot/P02458) | | 95995 | 9.14 |  |  | ✓ |
| gi|15149479 | alpha 1 type II collagen isoform 2, preproprotein | COL2A1 | [P02458](http://www.uniprot.org/uniprot/P02458) | | 27388 | 6.66 |  |  | ✓ |
| gi|18765746 | alpha 1 type XVIII collagen isoform 3 precursor | COL18A1 | [P39060](http://www.uniprot.org/uniprot/P39060) | | 175646 | 5.62 |  |  | ✓ |
| gi|4501883 | alpha 2 actin | ACTA2 | [P62736](http://www.uniprot.org/uniprot/P62736) | | 41748 | 5.24 |  |  | ✓ |
| gi|4504345 | alpha 2 globin | HBA1 | [P69905](http://www.uniprot.org/uniprot/P69905) | | 15117 | 8.73 | ✓ | ✓ | ✓ |
| gi|48762934 | alpha 2 type I collagen | COL1A2 | [P08123](http://www.uniprot.org/uniprot/P08123) | | 91700 | 10.05 | ✓ | ✓ | ✓ |
| gi|17402875 | alpha 2 type VI collagen isoform 2C2 precursor | COL6A2 | [P12110](http://www.uniprot.org/uniprot/P12110) | | 106444 | 5.85 | ✓ |  | ✓ |
| gi|17402877 | alpha 2 type VI collagen isoform 2C2a precursor | COL6A2 | [P12110](http://www.uniprot.org/uniprot/P12110) | | 106444 | 5.85 |  |  | ✓ |
| gi|55743098 | alpha 3 type VI collagen isoform 1 precursor | COL6A3 | [P12111](http://www.uniprot.org/uniprot/P12111) | | 340593 | 6.15 |  |  | ✓ |
| gi|55743102 | alpha 3 type VI collagen isoform 3 precursor | COL6A3 | [P12111](http://www.uniprot.org/uniprot/P12111) | | 340593 | 6.15 | ✓ |  |  |
| gi|55743104 | alpha 3 type VI collagen isoform 4 precursor | COL6A3 | [P12111](http://www.uniprot.org/uniprot/P12111) | | 340593 | 6.15 |  |  | ✓ |
| gi|55743106 | alpha 3 type VI collagen isoform 5 precursor | COL6A3 | [P12111](http://www.uniprot.org/uniprot/P12111) | | 340593 | 6.15 |  |  | ✓ |
| gi|38202257 | alpha glucosidase II alpha subunit isoform 2 | GANAB | [Q14697](http://www.uniprot.org/uniprot/Q14697) | | 103910 | 5.58 |  |  | ✓ |
| gi|21361399 | alpha isoform of regulatory subunit A, protein phosphatase 2 | PPP2R1A | [P30153](http://www.uniprot.org/uniprot/P30153) | | 65136 | 5 |  |  | ✓ |
| gi|4502067 | alpha-1-microglobulin/bikunin precursor | AMBP | [P02760](http://www.uniprot.org/uniprot/P02760) | | 20833 | 6.13 |  |  | ✓ |
| gi|4502337 | alpha-2-glycoprotein 1, zinc | AZGP1 | [P25311](http://www.uniprot.org/uniprot/P25311) | | 32125 | 5.58 |  |  | ✓ |
| gi|66932947 | alpha-2-macroglobulin precursor | A2M | [P01023](http://www.uniprot.org/uniprot/P01023) | | 160695 | 5.95 |  | ✓ | ✓ |
| gi|4502119 | amine oxidase, copper containing 3 precursor | AOC3 | [Q16853](http://www.uniprot.org/uniprot/Q16853) | | 84437 | 6.05 |  |  | ✓ |
| gi|4506061 | AMP-activated protein kinase, noncatalytic gamma-1 subunit isoform 1 | PRKAG1 | [P54619](http://www.uniprot.org/uniprot/P54619) | | 37556 | 6.42 |  |  | ✓ |
| gi|4502107 | annexin 5 | ANXA5 | [P08758](http://www.uniprot.org/uniprot/P08758) | | 35783 | 4.94 |  | ✓ | ✓ |
| gi|50845388 | annexin A2 isoform 1 | ANXA2 | [P07355](http://www.uniprot.org/uniprot/P07355) | | 38449 | 7.56 |  |  | ✓ |
| gi|4757756 | annexin A2 isoform 2 | ANXA2 | [P07355](http://www.uniprot.org/uniprot/P07355) | | 38449 | 7.56 | ✓ | ✓ | ✓ |
| gi|4502101 | annexin I | ANXA1 | [P04083](http://www.uniprot.org/uniprot/P04083) | | 38559 | 6.64 | ✓ | ✓ | ✓ |
| gi|4502105 | annexin IV | ANXA4 | [P09525](http://www.uniprot.org/uniprot/P09525) | | 35729 | 5.85 |  | ✓ | ✓ |
| gi|71773329 | annexin VI isoform 1 | ANXA6 | [P08133](http://www.uniprot.org/uniprot/P08133) | | 75695 | 5.42 |  |  | ✓ |
| gi|4557321 | apolipoprotein A-I preproprotein | APOA1 | [P02647](http://www.uniprot.org/uniprot/P02647) | | 28061 | 5.27 | ✓ | ✓ | ✓ |
| gi|4502149 | apolipoprotein A-II preproprotein | APOA2 | [P02652](http://www.uniprot.org/uniprot/P02652) | | 8702 | 5.05 |  |  | ✓ |
| gi|71773110 | apolipoprotein A-IV precursor | APOA4 | [P06727](http://www.uniprot.org/uniprot/P06727) | | 43376 | 5.18 |  | ✓ |  |
| gi|4502153 | apolipoprotein B precursor | APOB | [P04114](http://www.uniprot.org/uniprot/P04114) | | 512496 | 6.59 | ✓ |  | ✓ |
| gi|4502163 | apolipoprotein D precursor | APOD | [P05090](http://www.uniprot.org/uniprot/P05090) | | 19291 | 5.2 |  | ✓ |  |
| gi|4557327 | apolipoprotein H precursor | APOH | [P02749](http://www.uniprot.org/uniprot/P02749) | | 36231 | 8.37 |  |  | ✓ |
| gi|10947139 | arginase, type I | ARG1 | [P05089](http://www.uniprot.org/uniprot/P05089) | | 34713 | 6.72 |  |  | ✓ |
| gi|16950633 | argininosuccinate synthetase | ASS1 | [P00966](http://www.uniprot.org/uniprot/P00966) | | 46501 | 8.08 |  |  | ✓ |
| gi|45827806 | atlastin 3 | C18orf24 | [Q6DD88](http://www.uniprot.org/uniprot/Q6DD88) | | 60503 | 5.43 |  |  | ✓ |
| gi|21361565 | ATP synthase, H+ transporting, mitochondrial F0 complex, subunit b isoform 1 precursor | ATP5F1 | [P24539](http://www.uniprot.org/uniprot/P24539) | | 24610 | 9.1 |  | ✓ | ✓ |
| gi|6005717 | ATP synthase, H+ transporting, mitochondrial F0 complex, subunit E | ATP51 | [P56385](http://www.uniprot.org/uniprot/P56385) | | 7797 | 9.35 |  |  | ✓ |
| gi|50345982 | ATP synthase, H+ transporting, mitochondrial F1 complex, alpha subunit isoform b | | | | 58875 | 8.24 |  |  | ✓ |
| gi|4757810 | ATP synthase, H+ transporting, mitochondrial F1 complex, alpha subunit precursor | ATP5A1 | | [P25705](http://www.uniprot.org/uniprot/P25705) | 55175 | 8.28 | ✓ | ✓ | ✓ |
| gi|32189394 | ATP synthase, H+ transporting, mitochondrial F1 complex, beta subunit precursor | ATP5B | | [P06576](http://www.uniprot.org/uniprot/P06576) | 51737 | 5 | ✓ | ✓ | ✓ |
| gi|50345988 | ATP synthase, H+ transporting, mitochondrial F1 complex, gamma subunit isoform L (liver) precursor | ATP5C1 | | [P36542](http://www.uniprot.org/uniprot/P36542) | 30147 | 9.02 |  |  | ✓ |
| gi|19913428 | ATPase, H+ transporting, lysosomal 56/58kD, V1 subunit B, isoform 2 | ATP6V1B2 | | [P21281](http://www.uniprot.org/uniprot/P21281) | 56465 | 5.57 |  |  | ✓ |
| gi|19913424 | ATPase, H+ transporting, lysosomal 70kD, V1 subunit A, isoform 1 | ATP6V1A | | [P38606](http://www.uniprot.org/uniprot/P38606) | 68260 | 5.35 |  |  | ✓ |
| gi|51944966 | ATPase, H+/K+ exchanging, alpha polypeptide | ATP4A | | [P20648](http://www.uniprot.org/uniprot/P20648) | 113914 | 5.58 |  |  | ✓ |
| gi|55743069 | ATPase, H+/K+ transporting, nongastric, alpha polypeptide | | | | 126612 | 6.12 |  |  | ✓ |
| gi|10863945 | ATP-dependent DNA helicase II | XRCC5 | | [P13010](http://www.uniprot.org/uniprot/P13010) | 82521 | 5.55 | ✓ |  | ✓ |
| gi|4503841 | ATP-dependent DNA helicase II, 70 kDa subunit | XRCC6 | | [P12956](http://www.uniprot.org/uniprot/P12956) | 69668 | 6.23 |  |  | ✓ |
| gi|4501885 | beta actin | ACTB | | [P60709](http://www.uniprot.org/uniprot/P60709) | 41710 | 5.29 | ✓ |  | ✓ |
| gi|4504349 | beta globin | HBB | | [P68871](http://www.uniprot.org/uniprot/P68871) | 15857 | 6.81 | ✓ | ✓ | ✓ |
| gi|4504981 | beta-galactoside-binding lectin precursor | LGALS1 | | [P09382](http://www.uniprot.org/uniprot/P09382) | 14575 | 5.34 |  |  | ✓ |
| gi|4502403 | biglycan preproprotein | BGN | | [P21810](http://www.uniprot.org/uniprot/P21810) | 37218 | 8.13 |  |  | ✓ |
| gi|4502419 | biliverdin reductase B (flavin reductase (NADPH)) | BLVRB | | [P30043](http://www.uniprot.org/uniprot/P30043) | 21974 | 7.31 | ✓ |  | ✓ |
| gi|4557367 | bleomycin hydrolase | BLMH | | [Q13867](http://www.uniprot.org/uniprot/Q13867) | 52528 | 5.87 |  |  | ✓ |
| gi|21536286 | brain creatine kinase | CKB | | [P12277](http://www.uniprot.org/uniprot/P12277) | 42486 | 5.35 |  |  | ✓ |
| gi|21361370 | brain glycogen phosphorylase | PYGB | | [P11216](http://www.uniprot.org/uniprot/P11216) | 96503 | 6.41 |  |  | ✓ |
| gi|50511945 | cajalin 2 isoform a | ANKS1B | | [Q7Z6G8](http://www.uniprot.org/uniprot/Q7Z6G8) | 137968 | 5.94 |  |  | ✓ |
| gi|4502549 | calmodulin 2 | CALM2 | | [P62158](http://www.uniprot.org/uniprot/P62158) | 16696 | 4.09 |  |  | ✓ |
| gi|4885111 | calmodulin-like 3 | CALML3 | | [P27482](http://www.uniprot.org/uniprot/P27482) | 16749 | 4.3 |  |  | ✓ |
| gi|8393159 | calmodulin-like skin protein | CALML5 | | [Q9NZT1](http://www.uniprot.org/uniprot/Q9NZT1) | 15752 | 4.34 |  |  | ✓ |
| gi|10716563 | calnexin precursor | CANX | | [P27824](http://www.uniprot.org/uniprot/P27824) | 65355 | 4.47 |  |  | ✓ |
| gi|4502565 | calpain, small subunit 1 | CAPNS1 | | [P04632](http://www.uniprot.org/uniprot/P04632) | 28298 | 5.05 |  |  | ✓ |
| gi|27765083 | calpastatin isoform a |  | | [P20810](http://www.uniprot.org/uniprot/P20810) | 76526 | 4.98 |  |  | ✓ |
| gi|27765085 | calpastatin isoform b | CAST | | [P20810](http://www.uniprot.org/uniprot/P20810) | 76526 | 4.98 |  |  | ✓ |
| gi|21361120 | calponin 1, basic, smooth muscle | CNN1 | | [P51911](http://www.uniprot.org/uniprot/P51911) | 33150 | 9.14 |  |  | ✓ |
| gi|4757900 | calreticulin precursor | CALR | | [P27797](http://www.uniprot.org/uniprot/P27797) | 46438 | 4.29 |  |  | ✓ |
| gi|4502517 | carbonic anhydrase I | CA1 | | [P00915](http://www.uniprot.org/uniprot/P00915) | 28721 | 6.63 |  | ✓ | ✓ |
| gi|4557395 | carbonic anhydrase II | CA2 | | [P00918](http://www.uniprot.org/uniprot/P00918) | 29097 | 6.86 |  |  | ✓ |
| gi|4502599 | carbonyl reductase 1 | SETD4 | | [P16152](http://www.uniprot.org/uniprot/P16152) | 30225 | 8.55 |  |  | ✓ |
| gi|4502601 | carbonyl reductase 3 | CBR3 | | [O75828](http://www.uniprot.org/uniprot/O75828) | 30700 | 5.82 |  |  | ✓ |
| gi|4885049 | cardiac muscle alpha actin proprotein | ACTC1 | | [P68032](http://www.uniprot.org/uniprot/P68032) | 41758 | 5.23 |  |  | ✓ |
| gi|51944962 | cartilage intermediate layer protein | CILP | | [O75339](http://www.uniprot.org/uniprot/O75339) | 130275 | 8.74 | ✓ |  |  |
| gi|6912286 | caspase 14 precursor | CASP14 | | [P31944](http://www.uniprot.org/uniprot/P31944) | 30342 | 5.44 |  | ✓ | ✓ |
| gi|4557014 | catalase | CAT | | [P04040](http://www.uniprot.org/uniprot/P04040) | 59588 | 6.95 |  | ✓ | ✓ |
| gi|4503131 | catenin (cadherin-associated protein), beta 1, 88kDa | CTNNB1 | | [P35222](http://www.uniprot.org/uniprot/P35222) | 85442 | 5.53 |  |  | ✓ |
| gi|10835010 | catenin (cadherin-associated protein), delta 1 | CTNND1 | | [O60716](http://www.uniprot.org/uniprot/O60716) | 108103 | 5.86 |  |  | ✓ |
| gi|4503141 | cathepsin C isoform a preproprotein | CTSC | | [P53634](http://www.uniprot.org/uniprot/P53634) | 12587 | 4.94 |  |  | ✓ |
| gi|4503143 | cathepsin D preproprotein | CTSD | | [P07339](http://www.uniprot.org/uniprot/P07339) | 37828 | 5.6 | ✓ |  | ✓ |
| gi|4503149 | cathepsin G preproprotein | CTSG | | [P08311](http://www.uniprot.org/uniprot/P08311) | 26741 | 11.37 | ✓ |  |  |
| gi|15451856 | caveolin 1 | CAV1 | | [Q03135](http://www.uniprot.org/uniprot/Q03135) | 20327 | 5.64 |  |  | ✓ |
| gi|48255935 | CD44 antigen isoform 1 precursor | CD44 | | [P16070](http://www.uniprot.org/uniprot/P16070) | 79178 | 5.1 |  |  | ✓ |
| gi|5901922 | CDC37 homolog | CDC37 | | [Q16543](http://www.uniprot.org/uniprot/Q16543) | 44440 | 5.17 |  | ✓ |  |
| gi|4757952 | cell division cycle 42 isoform 1 | CDC42 | | [P60953](http://www.uniprot.org/uniprot/P60953) | 20934 | 5.76 |  |  | ✓ |
| gi|51890223 | centrosome protein cep290 | CEP290 | | [O15078](http://www.uniprot.org/uniprot/O15078) | 290207 | 5.75 |  |  | ✓ |
| gi|4557485 | ceruloplasmin (ferroxidase) | CP | | [P00450](http://www.uniprot.org/uniprot/P00450) | 120009 | 5.41 |  |  | ✓ |
| gi|31542947 | chaperonin | HSPD1 | | [P10809](http://www.uniprot.org/uniprot/P10809) | 57927 | 5.24 |  |  | ✓ |
| gi|5453603 | chaperonin containing TCP1, subunit 2 | CCT2 | | [P78371](http://www.uniprot.org/uniprot/P78371) | 57321 | 6.02 |  |  | ✓ |
| gi|58761486 | chaperonin containing TCP1, subunit 3 isoform b | CCT3 | | [P49368](http://www.uniprot.org/uniprot/P49368) | 60495 | 6.1 |  |  | ✓ |
| gi|58761484 | chaperonin containing TCP1, subunit 3 isoform c | CCT3 | | [P49368](http://www.uniprot.org/uniprot/P49368) | 60495 | 6.1 |  |  | ✓ |
| gi|4502643 | chaperonin containing TCP1, subunit 6A isoform a | CCT6A | | [P40227](http://www.uniprot.org/uniprot/P40227) | 57857 | 6.25 |  |  | ✓ |
| gi|5453607 | chaperonin containing TCP1, subunit 7 isoform a | CCT7 | | [Q99832](http://www.uniprot.org/uniprot/Q99832) | 59329 | 7.55 |  |  | ✓ |
| gi|14251209 | chloride intracellular channel 1 | CLIC1 | | [O00299](http://www.uniprot.org/uniprot/O00299) | 26775 | 5.09 |  |  | ✓ |
| gi|24308201 | chromosome 20 open reading frame 3 | C20orf3 | | [Q9HDC9](http://www.uniprot.org/uniprot/Q9HDC9) | 46451 | 5.82 | ✓ |  |  |
| gi|4502907 | chymase 1, mast cell preproprotein | CMA1 | | [P23946](http://www.uniprot.org/uniprot/P23946) | 25014 | 9.6 | ✓ |  |  |
| gi|45545437 | citrate lyase beta like | CLYBL | | [Q8N0X4](http://www.uniprot.org/uniprot/Q8N0X4) | 34992 | 6.62 |  |  | ✓ |
| gi|38327625 | citrate synthase precursor, isoform a | CS | | [O75390](http://www.uniprot.org/uniprot/O75390) | 48975 | 7.39 |  |  | ✓ |
| gi|4501929 | class I alcohol dehydrogenase, alpha subunit | ADH1A | | [P07327](http://www.uniprot.org/uniprot/P07327) | 39701 | 8.26 |  |  | ✓ |
| gi|4758012 | clathrin heavy chain 1 | CLTC | | [Q00610](http://www.uniprot.org/uniprot/Q00610) | 191361 | 5.48 |  |  | ✓ |
| gi|9257202 | clathrin, heavy polypeptide-like 1 isoform b | CLTCL1 | | [P53675](http://www.uniprot.org/uniprot/P53675) | 186910 | 5.57 | ✓ |  | ✓ |
| gi|42716297 | clusterin isoform 1 | CLU | | [P10909](http://www.uniprot.org/uniprot/P10909) | 50031 | 5.89 |  | ✓ | ✓ |
| gi|8922699 | CNDP dipeptidase 2 (metallopeptidase M20 family) | CNDP2 | | [Q96KP4](http://www.uniprot.org/uniprot/Q96KP4) | 52714 | 5.66 |  |  | ✓ |
| gi|4503631 | coagulation factor XIII A1 subunit precursor | F13A1 | | [P00488](http://www.uniprot.org/uniprot/P00488) | 79195 | 5.81 |  |  | ✓ |
| gi|4758030 | coatomer protein complex, subunit alpha | COPA | | [P53621](http://www.uniprot.org/uniprot/P53621) | 138258 | 7.7 |  |  | ✓ |
| gi|5031635 | cofilin 1 (non-muscle) | CFL1 | | [P23528](http://www.uniprot.org/uniprot/P23528) | 18360 | 8.26 |  |  | ✓ |
| gi|4502847 | cold inducible RNA binding protein | CIRBP | | [Q14011](http://www.uniprot.org/uniprot/Q14011) | 18637 | 9.51 |  |  | ✓ |
| gi|15011913 | collagen, type VI, alpha 1 precursor | COL6A1 | | [P12109](http://www.uniprot.org/uniprot/P12109) | 106412 | 5.2 | ✓ |  | ✓ |
| gi|55743096 | collagen, type XIV, alpha 1 | COL14A1 | | [Q05707](http://www.uniprot.org/uniprot/Q05707) | 189952 | 5.1 |  |  | ✓ |
| gi|4557385 | complement component 3 precursor | C3 | | [P01024](http://www.uniprot.org/uniprot/P01024) | 184835 | 6 |  |  | ✓ |
| gi|67190748 | complement component 4A preproprotein | C4A | | [P0C0L4](http://www.uniprot.org/uniprot/P0C0L4) | 71634 | 8.69 |  |  | ✓ |
| gi|67782351 | complement component 4B preproprotein | C4B | | [P0C0L5](http://www.uniprot.org/uniprot/P0C0L5) | 71634 | 8.69 |  |  | ✓ |
| gi|4557391 | complement component 8, beta polypeptide preproprotein | C8B | | [P07358](http://www.uniprot.org/uniprot/P07358) | 61003 | 7.85 |  |  | ✓ |
| gi|67782358 | complement factor B preproprotein | CFB | | [P00751](http://www.uniprot.org/uniprot/P00751) | 82948 | 6.66 |  |  | ✓ |
| gi|42544239 | complement factor D preproprotein | CFD | | [P00746](http://www.uniprot.org/uniprot/P00746) | 24389 | 6.85 |  |  | ✓ |
| gi|62739186 | complement factor H isoform a precursor | CFH | | [P08603](http://www.uniprot.org/uniprot/P08603) | 136963 | 6.12 |  |  | ✓ |
| gi|25141323 | copine V | CPNE5 | | [Q9HCH3](http://www.uniprot.org/uniprot/Q9HCH3) | 65692 | 5.65 |  |  | ✓ |
| gi|10334859 | creatine kinase, mitochondrial 1B precursor | CKMT1B | | [P12532](http://www.uniprot.org/uniprot/P12532) | 43053 | 7.31 |  |  | ✓ |
| gi|4507557 | C-type lectin domain family 3, member B | CLEC3B | | [P05452](http://www.uniprot.org/uniprot/P05452) | 20156 | 5.8 |  |  | ✓ |
| gi|4503117 | cystatin B | CSTB | | [P04080](http://www.uniprot.org/uniprot/P04080) | 11133 | 6.96 |  |  | ✓ |
| gi|4758086 | cysteine and glycine-rich protein 1 | CSRP1 | | [P21291](http://www.uniprot.org/uniprot/P21291) | 20423 | 8.92 |  |  | ✓ |
| gi|4503183 | cytochrome b-5 isoform 2 | CYB5A | | [P00167](http://www.uniprot.org/uniprot/P00167) | 15189 | 4.88 |  | ✓ |  |
| gi|4503327 | cytochrome b5 reductase isoform 1 | CYB5R3 | | [P00387](http://www.uniprot.org/uniprot/P00387) | 34082 | 7.31 |  |  | ✓ |
| gi|17999528 | cytochrome c oxidase subunit VIa polypeptide 1 precursor | COX6A1 | | [P12074](http://www.uniprot.org/uniprot/P12074) | 9613 | 6.42 |  |  | ✓ |
| gi|7705545 | cytokeratin 2 | KRT76 | | [Q01546](http://www.uniprot.org/uniprot/Q01546) | 65830 | 8.38 | ✓ |  | ✓ |
| gi|4758618 | cytokeratin type II | KRT75 | | [O95678](http://www.uniprot.org/uniprot/O95678) | 59524 | 7.6 | ✓ |  | ✓ |
| gi|19920317 | cytoskeleton-associated protein 4 | CKAP4 | | [Q07065](http://www.uniprot.org/uniprot/Q07065) | 65983 | 5.63 |  |  | ✓ |
| gi|5174539 | cytosolic malate dehydrogenase | MDH1 | | [P40925](http://www.uniprot.org/uniprot/P40925) | 36272 | 6.89 |  |  | ✓ |
| gi|4826686 | DEAD (Asp-Glu-Ala-Asp) box polypeptide 1 | DDX1 | | [Q92499](http://www.uniprot.org/uniprot/Q92499) | 82380 | 6.8 |  |  | ✓ |
| gi|21040371 | DEAD (Asp-Glu-Ala-Asp) box polypeptide 39 isoform 1 | DDX39 | | [O00148](http://www.uniprot.org/uniprot/O00148) | 49098 | 5.46 |  |  | ✓ |
| gi|4503271 | decorin isoform a preproprotein | DCN | | [P07585](http://www.uniprot.org/uniprot/P07585) | 36304 | 8.76 | ✓ |  | ✓ |
| gi|4504351 | delta globin | HBD | | [P02042](http://www.uniprot.org/uniprot/P02042) | 15914 | 7.97 | ✓ | ✓ | ✓ |
| gi|32307172 | dermatopontin precursor | DPT | | [Q07507](http://www.uniprot.org/uniprot/Q07507) | 21950 | 4.78 |  |  | ✓ |
| gi|16751921 | dermcidin precursor | DCD | | [P81605](http://www.uniprot.org/uniprot/P81605) | 9254 | 5.64 |  |  | ✓ |
| gi|55749932 | desmin | DES | | [P17661](http://www.uniprot.org/uniprot/P17661) | 53372 | 5.21 | ✓ | ✓ | ✓ |
| gi|4826702 | desmocollin 1 isoform Dsc1b preproprotein | DSC1 | | [Q08554](http://www.uniprot.org/uniprot/Q08554) | 84932 | 4.89 |  |  | ✓ |
| gi|4503399 | desmocollin 3 isoform Dsc3a preproprotein | DSC3 | | [Q14574](http://www.uniprot.org/uniprot/Q14574) | 84663 | 5.02 |  |  | ✓ |
| gi|4503401 | desmoglein 1 preproprotein | DSG1 | | [Q02413](http://www.uniprot.org/uniprot/Q02413) | 107636 | 4.77 |  |  | ✓ |
| gi|58530840 | desmoplakin isoform I | DSP | | [P15924](http://www.uniprot.org/uniprot/P15924) | 331569 | 6.44 | ✓ |  | ✓ |
| gi|58530842 | desmoplakin isoform II | DSP | | [P15924](http://www.uniprot.org/uniprot/P15924) | 331569 | 6.44 |  |  | ✓ |
| gi|22027638 | desmuslin isoform A | [SYNM](http://www.uniprot.org/uniprot/O15061) | | [O15061](http://www.uniprot.org/uniprot/O15061) | 172663 | 5.09 |  |  | ✓ |
| gi|5802966 | destrin isoform a | DSTN | | [P60981](http://www.uniprot.org/uniprot/P60981) | 18362 | 8.12 |  |  | ✓ |
| gi|10140853 | diazepam binding inhibitor | DBI | | [P07108](http://www.uniprot.org/uniprot/P07108) | 9907 | 6.11 |  |  | ✓ |
| gi|7705925 | dicarbonyl/L-xylulose reductase | DCXR | | [Q7Z4W1](http://www.uniprot.org/uniprot/Q7Z4W1) | 25897 | 8.33 | ✓ |  |  |
| gi|31543380 | DJ-1 protein | PARK7 | | [Q99497](http://www.uniprot.org/uniprot/Q99497) | 19878 | 6.33 |  | ✓ | ✓ |
| gi|21359935 | Down syndrome cell adhesion molecule like 1 | DSCAML1 | | [Q8TD84](http://www.uniprot.org/uniprot/Q8TD84) | 222189 | 8.39 |  |  | ✓ |
| gi|56549119 | dynamin 2 isoform 3 | DNM2 | | [P50570](http://www.uniprot.org/uniprot/P50570) | 98003 | 7.04 |  |  | ✓ |
| gi|24308169 | dynein, axonemal, heavy polypeptide 3 | ZNF571 | | [Q8TD57](http://www.uniprot.org/uniprot/Q8TD57) | 470468 | 6.04 |  |  | ✓ |
| gi|33350932 | dynein, cytoplasmic, heavy polypeptide 1 | DYNC1H1 | | [Q14204](http://www.uniprot.org/uniprot/Q14204) | 532072 | 6.01 |  |  | ✓ |
| gi|4503393 | dystrophin related protein 2 | DRP2 | | [Q13474](http://www.uniprot.org/uniprot/Q13474) | 107894 | 5.82 |  |  | ✓ |
| gi|21361462 | EH-domain containing 2 | EHD2 | | [Q9NZN4](http://www.uniprot.org/uniprot/Q9NZN4) | 61123 | 6.03 |  |  | ✓ |
| gi|7657056 | EH-domain containing 3 | EHD3 | | [Q9NZN3](http://www.uniprot.org/uniprot/Q9NZN3) | 61857 | 6.06 |  |  | ✓ |
| gi|9558749 | elongation factor-2 kinase | EEF2K | | [O00418](http://www.uniprot.org/uniprot/O00418) | 82120 | 5.19 |  |  | ✓ |
| gi|4503445 | endothelial cell growth factor 1 (platelet-derived) | ECGF1 | | [P19971](http://www.uniprot.org/uniprot/P19971) | 48994 | 5.36 |  |  | ✓ |
| gi|4503571 | enolase 1 | ENO1 | | [P06733](http://www.uniprot.org/uniprot/P06733) | 47008 | 6.99 | ✓ | ✓ | ✓ |
| gi|5803011 | enolase 2 | ENO2 | | [P09104](http://www.uniprot.org/uniprot/P09104) | 47108 | 4.91 | ✓ |  | ✓ |
| gi|4503573 | enolase 3 | ENO3 | | [P13929](http://www.uniprot.org/uniprot/P13929) | 46826 | 7.73 |  | ✓ | ✓ |
| gi|27597073 | epoxide hydrolase 2, cytoplasmic | EPHX2 | | [P34913](http://www.uniprot.org/uniprot/P34913) | 62575 | 5.91 |  |  | ✓ |
| gi|4885393 | epsilon globin | HBE1 | | [P02100](http://www.uniprot.org/uniprot/P02100) | 16061 | 8.69 |  |  | ✓ |
| gi|7657069 | ERO1-like | ERO1L | | [Q96HE7](http://www.uniprot.org/uniprot/Q96HE7) | 51959 | 5.37 |  |  | ✓ |
| gi|33413400 | esterase D/formylglutathione hydrolase | ESD | | [P10768](http://www.uniprot.org/uniprot/P10768) | 31442 | 6.54 |  |  | ✓ |
| gi|4503471 | eukaryotic translation elongation factor 1 alpha 1 | EEF1A1 | | [P68104](http://www.uniprot.org/uniprot/P68104) | 50109 | 9.1 | ✓ | ✓ | ✓ |
| gi|4503483 | eukaryotic translation elongation factor 2 | EEF2 | | [P13639](http://www.uniprot.org/uniprot/P13639) | 95146 | 6.42 |  |  | ✓ |
| gi|10801345 | eukaryotic translation initiation factor 3, subunit 12 | EIF3S12 | | [Q9UBQ5](http://www.uniprot.org/uniprot/Q9UBQ5) | 24912 | 4.81 |  |  | ✓ |
| gi|4503519 | eukaryotic translation initiation factor 3, subunit 5 epsilon, 47kDa | EIF3S5 | | [O00303](http://www.uniprot.org/uniprot/O00303) | 37409 | 5.24 |  |  | ✓ |
| gi|4503529 | eukaryotic translation initiation factor 4A, isoform 1 | EIF4A1 | | [P60842](http://www.uniprot.org/uniprot/P60842) | 46125 | 5.32 |  |  | ✓ |
| gi|4503531 | eukaryotic translation initiation factor 4A, isoform 2 | EIF4A2 | | [Q14240](http://www.uniprot.org/uniprot/Q14240) | 46373 | 5.33 |  |  | ✓ |
| gi|153791632 | eukaryotic translation initiation factor 5A-like 1 | EIF5AL1 | | [Q6IS14](http://www.uniprot.org/uniprot/Q6IS14) | 16762 | 4.85 |  |  | ✓ |
| gi|4826659 | F-actin capping protein beta subunit | CAPZB | | [P47756](http://www.uniprot.org/uniprot/P47756) | 31200 | 5.36 |  |  | ✓ |
| gi|4507115 | fascin 1 | FSCN1 | | [Q16658](http://www.uniprot.org/uniprot/Q16658) | 54365 | 6.81 |  |  | ✓ |
| gi|4507617 | fast skeletal muscle troponin C | TNNC2 | | [P02585](http://www.uniprot.org/uniprot/P02585) | 17979 | 4.06 |  | ✓ |  |
| gi|4557579 | fatty acid binding protein 4, adipocyte | FABP4 | | [P15090](http://www.uniprot.org/uniprot/P15090) | 14578 | 6.8 |  |  | ✓ |
| gi|41872631 | fatty acid synthase | FASN | | [P49327](http://www.uniprot.org/uniprot/P49327) | 273227 | 5.99 | ✓ |  | ✓ |
| gi|24430141 | fibrillin 1 | FBN1 | | [P35555](http://www.uniprot.org/uniprot/P35555) | 309198 | 4.79 |  |  | ✓ |
| gi|11761629 | fibrinogen, alpha chain isoform alpha preproprotein | FGA | | [P02671](http://www.uniprot.org/uniprot/P02671) | 1536 | 3.92 |  |  | ✓ |
| gi|4503689 | fibrinogen, alpha chain isoform alpha-E preproprotein | FGA | | [P02671](http://www.uniprot.org/uniprot/P02671) | 91303 | 5.79 | ✓ |  | ✓ |
| gi|70906435 | fibrinogen, beta chain preproprotein | FGB | | [P02675](http://www.uniprot.org/uniprot/P02675) | 50731 | 7.95 | ✓ |  | ✓ |
| gi|70906437 | fibrinogen, gamma chain isoform gamma-A precursor | FGG | | [P02679](http://www.uniprot.org/uniprot/P02679) | 48452 | 5.24 | ✓ |  | ✓ |
| gi|71040111 | fibromodulin precursor | FMOD | | [Q06828](http://www.uniprot.org/uniprot/Q06828) | 41191 | 5.66 |  |  | ✓ |
| gi|16933542 | fibronectin 1 isoform 3 preproprotein | FN1 | | [P02751](http://www.uniprot.org/uniprot/P02751) | 19607 | 3.84 |  |  | ✓ |
| gi|34734066 | fibulin 1 isoform D | FBLN1 | | [P23142](http://www.uniprot.org/uniprot/P23142) | 74241 | 5.03 |  |  | ✓ |
| gi|51873053 | fibulin 2 precursor, isoform a | FBLN2 | | [Q86V58](http://www.uniprot.org/uniprot/Q86V58) | 126425 | 4.73 |  |  | ✓ |
| gi|60097902 | filaggrin | FLG | | [P20930](http://www.uniprot.org/uniprot/P20930) | 434922 | 9.24 |  |  | ✓ |
| gi|62122917 | filaggrin 2 | RP1 | | [Q5D862](http://www.uniprot.org/uniprot/Q5D862) | 247928 | 8.45 |  |  | ✓ |
| gi|4503745 | filamin 1 | FLNA | | [P21333](http://www.uniprot.org/uniprot/P21333) | 280433 | 5.7 | ✓ |  | ✓ |
| gi|4503747 | filamin B, beta | FLNB | | [O75369](http://www.uniprot.org/uniprot/O75369) | 278021 | 5.49 | ✓ |  | ✓ |
| gi|190341068 | forkhead-associated (FHA) phosphopeptide binding domain 1 | FHAD1 | | [B1AJZ9](http://www.uniprot.org/uniprot/B1AJZ9) | 161804 | 6.52 |  |  | ✓ |
| gi|4885063 | fructose-bisphosphate aldolase C | ALDOC | | [P09972](http://www.uniprot.org/uniprot/P09972) | 39300 | 6.46 |  |  | ✓ |
| gi|4504983 | galectin 3 | LGALS3 | | [P17931](http://www.uniprot.org/uniprot/P17931) | 26005 | 8.6 |  |  | ✓ |
| gi|4504985 | galectin 7 | LGALS7 | | [P47929](http://www.uniprot.org/uniprot/P47929) | 14935 | 7 | ✓ | ✓ | ✓ |
| gi|66279526 | gamma filamin | FLNC | | [Q14315](http://www.uniprot.org/uniprot/Q14315) | 290778 | 5.68 | ✓ |  | ✓ |
| gi|4503987 | gamma-glutamyl hydrolase precursor | GGH | | [Q92820](http://www.uniprot.org/uniprot/Q92820) | 33626 | 7.19 |  |  | ✓ |
| gi|30039702 | gasdermin 1 | GSDM1 | | [Q96QA5](http://www.uniprot.org/uniprot/Q96QA5) | 49348 | 5.19 |  |  | ✓ |
| gi|4503971 | GDP dissociation inhibitor 1 | GDI1 | | [P31150](http://www.uniprot.org/uniprot/P31150) | 50550 | 5 |  |  | ✓ |
| gi|6598323 | GDP dissociation inhibitor 2 | GDI2 | | [P50395](http://www.uniprot.org/uniprot/P50395) | 50631 | 6.11 |  |  | ✓ |
| gi|4504165 | gelsolin isoform a | GSN | | [P06396](http://www.uniprot.org/uniprot/P06396) | 82908 | 5.72 |  |  | ✓ |
| gi|38044288 | gelsolin isoform b | GSN | | [P06396](http://www.uniprot.org/uniprot/P06396) | 82908 | 5.72 |  |  | ✓ |
| gi|6715607 | G-gamma globin | HBG2 | | [P69892](http://www.uniprot.org/uniprot/P69892) | 15985 | 6.71 | ✓ |  | ✓ |
| gi|4503979 | glial fibrillary acidic protein | GFAP | | [P14136](http://www.uniprot.org/uniprot/P14136) | 49850 | 5.42 | ✓ |  | ✓ |
| gi|4557619 | glucan (1,4-alpha-), branching enzyme 1 | GBE1 | | [Q04446](http://www.uniprot.org/uniprot/Q04446) | 80409 | 5.87 |  |  | ✓ |
| gi|18201905 | glucose phosphate isomerase | GPI | | [P06744](http://www.uniprot.org/uniprot/P06744) | 62976 | 8.44 |  | ✓ | ✓ |
| gi|31377775 | glutamate dehydrogenase 2 | GLUD2 | | [P49448](http://www.uniprot.org/uniprot/P49448) | 56018 | 6.74 |  |  | ✓ |
| gi|23065552 | glutathione S-transferase M3 | GSTM3 | | [P21266](http://www.uniprot.org/uniprot/P21266) | 26411 | 5.37 |  |  | ✓ |
| gi|4504169 | glutathione synthetase | GSS | | [P48637](http://www.uniprot.org/uniprot/P48637) | 52352 | 5.67 |  |  | ✓ |
| gi|4504183 | glutathione transferase | GSTP1 | | [P09211](http://www.uniprot.org/uniprot/P09211) | 23210 | 5.44 |  | ✓ | ✓ |
| gi|7669492 | glyceraldehyde-3-phosphate dehydrogenase | GAPDH | | [P04406](http://www.uniprot.org/uniprot/P04406) | 35899 | 8.58 | ✓ | ✓ | ✓ |
| gi|4505405 | glycoprotein (transmembrane) nmb isoform b precursor | GPNMB | | [Q14956](http://www.uniprot.org/uniprot/Q14956) | 61469 | 6.29 |  |  | ✓ |
| gi|5729842 | glyoxalase I | GLO1 | | [Q04760](http://www.uniprot.org/uniprot/Q04760) | 20633 | 5.12 |  |  | ✓ |
| gi|6912396 | glyoxylate reductase/hydroxypyruvate reductase | GRHPR | | [Q9UBQ7](http://www.uniprot.org/uniprot/Q9UBQ7) | 35646 | 7.01 |  |  | ✓ |
| gi|19923483 | GTPase Rab14 | RAB14 | | [P61106](http://www.uniprot.org/uniprot/P61106) | 23751 | 5.86 |  |  | ✓ |
| gi|42476111 | guanine nucleotide binding protein (G protein) alpha 12 | GNA12 | | [Q03113](http://www.uniprot.org/uniprot/Q03113) | 44120 | 9.84 |  |  | ✓ |
| gi|4504043 | guanine nucleotide binding protein (G protein), alpha activating activity polypeptide, olfactory type isoform 2 | GNAL | | [P38405](http://www.uniprot.org/uniprot/P38405) | 44149 | 6.25 |  |  | ✓ |
| gi|33946324 | guanine nucleotide binding protein (G protein), alpha inhibiting activity polypeptide 1 | GNAI1 | | [P63096](http://www.uniprot.org/uniprot/P63096) | 40204 | 5.7 |  |  | ✓ |
| gi|4504041 | guanine nucleotide binding protein (G protein), alpha inhibiting activity polypeptide 2 | GNAI2 | | [P04899](http://www.uniprot.org/uniprot/P04899) | 40294 | 5.34 |  |  | ✓ |
| gi|5729850 | guanine nucleotide binding protein (G protein), alpha inhibiting activity polypeptide 3 | GNAI3 | | [P08754](http://www.uniprot.org/uniprot/P08754) | 40375 | 5.51 |  |  | ✓ |
| gi|4504047 | guanine nucleotide binding protein, alpha stimulating activity polypeptide 1 isoform a | GNAS | | [P63092](http://www.uniprot.org/uniprot/P63092) | 45505 | 5.59 |  |  | ✓ |
| gi|18426898 | guanine nucleotide binding protein, alpha stimulating activity polypeptide 1 isoform c | GNAS | | [P63092](http://www.uniprot.org/uniprot/P63092) | 45505 | 5.59 |  |  | ✓ |
| gi|20330805 | guanine nucleotide binding protein, alpha transducing activity polypeptide 2 | GNAT2 | | [P19087](http://www.uniprot.org/uniprot/P19087) | 40019 | 5.11 |  |  | ✓ |
| gi|38788319 | guanine nucleotide binding protein-like 1 | GNL1 | | [P36915](http://www.uniprot.org/uniprot/P36915) | 68619 | 5.57 |  |  | ✓ |
| gi|11055998 | guanine nucleotide-binding protein, beta-4 subunit | GNB4 | | [Q9HAV0](http://www.uniprot.org/uniprot/Q9HAV0) | 37412 | 5.59 |  |  | ✓ |
| gi|4885375 | H1 histone family, member 2 | HIST1H1C | | [P16403](http://www.uniprot.org/uniprot/P16403) | 21221 | 10.94 |  |  | ✓ |
| gi|4885377 | H1 histone family, member 3 | HIST1H1D | | [P16402](http://www.uniprot.org/uniprot/P16402) | 22205 | 11.02 |  |  | ✓ |
| gi|4885379 | H1 histone family, member 4 | HIST1H1E | | [P10412](http://www.uniprot.org/uniprot/P10412) | 21721 | 11.03 |  |  | ✓ |
| gi|4504239 | H2A histone family, member C | HIST1H2AI | | [P0C0S8](http://www.uniprot.org/uniprot/P0C0S8) | 13952 | 10.9 | ✓ |  |  |
| gi|4504251 | H2A histone family, member O | HIST2H2AA3 | | [Q6FI13](http://www.uniprot.org/uniprot/Q6FI13) | 13956 | 10.9 | ✓ |  |  |
| gi|4504255 | H2A histone family, member Z | H2AFZ | | [P0C0S5](http://www.uniprot.org/uniprot/P0C0S5) | 13414 | 10.58 | ✓ |  |  |
| gi|4504257 | H2B histone family, member A | HIST1H2BC | | [P62807](http://www.uniprot.org/uniprot/P62807) | 13679 | 10.32 | ✓ |  | ✓ |
| gi|10800138 | H2B histone family, member B | HIST1H2BD | | [P58876](http://www.uniprot.org/uniprot/P58876) | 13797 | 10.32 |  |  | ✓ |
| gi|4504259 | H2B histone family, member C | HIST1H2BL | | [Q99880](http://www.uniprot.org/uniprot/Q99880) | 13813 | 10.32 |  |  | ✓ |
| gi|4504263 | H2B histone family, member E | HIST1H2BM | | [Q99879](http://www.uniprot.org/uniprot/Q99879) | 13850 | 10.32 |  |  | ✓ |
| gi|4504301 | H4 histone family, member A | HIST1H4A | | [P62805](http://www.uniprot.org/uniprot/P62805) | 11229 | 11.36 | ✓ |  | ✓ |
| gi|14149777 | haloacid dehalogenase-like hydrolase domain containing 2 | HDHD2 | | [Q9H0R4](http://www.uniprot.org/uniprot/Q9H0R4) | 28518 | 5.84 |  |  | ✓ |
| gi|4826762 | haptoglobin | HP | | [P00738](http://www.uniprot.org/uniprot/P00738) | 43322 | 6.13 |  | ✓ | ✓ |
| gi|4504523 | heat shock 10kDa protein 1 | HSPE1 | | [P61604](http://www.uniprot.org/uniprot/P61604) | 10794 | 8.91 |  |  | ✓ |
| gi|4504517 | heat shock 27kDa protein 1 | HSPB1 | | [P04792](http://www.uniprot.org/uniprot/P04792) | 22768 | 5.98 | ✓ | ✓ | ✓ |
| gi|19923193 | heat shock 70kD protein binding protein | ST13 | | [P50502](http://www.uniprot.org/uniprot/P50502) | 41305 | 5.18 |  |  | ✓ |
| gi|5123454 | heat shock 70kDa protein 1A | HSPA1A | | [P08107](http://www.uniprot.org/uniprot/P08107) | 70009 | 5.48 |  |  | ✓ |
| gi|4885431 | heat shock 70kDa protein 1B | HSPA1B | | [P08107](http://www.uniprot.org/uniprot/P08107) | 70009 | 5.48 |  |  | ✓ |
| gi|13676857 | heat shock 70kDa protein 2 | HSPA2 | | [P54652](http://www.uniprot.org/uniprot/P54652) | 69978 | 5.56 |  |  | ✓ |
| gi|16507237 | heat shock 70kDa protein 5 | HSPA5 | | [P11021](http://www.uniprot.org/uniprot/P11021) | 70435 | 5.01 |  |  | ✓ |
| gi|34419635 | heat shock 70kDa protein 6 | HSPA6 | | [P17066](http://www.uniprot.org/uniprot/P17066) | 70984 | 5.81 |  |  | ✓ |
| gi|5729877 | heat shock 70kDa protein 8 isoform 1 | HSPA8 | | [P11142](http://www.uniprot.org/uniprot/P11142) | 70723 | 5.37 |  |  | ✓ |
| gi|24234688 | heat shock 70kDa protein 9B precursor | HSPA9 | | [P38646](http://www.uniprot.org/uniprot/P38646) | 68716 | 5.44 |  |  | ✓ |
| gi|20149594 | heat shock 90kDa protein 1, beta | HSP90AB1 | | [P08238](http://www.uniprot.org/uniprot/P08238) | 83081 | 4.97 |  |  | ✓ |
| gi|40254816 | heat shock protein 90kDa alpha (cytosolic), class A member 1 isoform 2 | HSP90AA1 | | [P07900](http://www.uniprot.org/uniprot/P07900) | 84476 | 4.94 |  |  | ✓ |
| gi|7657603 | heme binding protein 2 | HEBP2 | | [Q9Y5Z4](http://www.uniprot.org/uniprot/Q9Y5Z4) | 22861 | 4.58 |  | ✓ | ✓ |
| gi|11321561 | hemopexin | HPX | | [P02790](http://www.uniprot.org/uniprot/P02790) | 49264 | 6.43 |  |  | ✓ |
| gi|4504445 | heterogeneous nuclear ribonucleoprotein A1 isoform a | HNRPA1 | | [P09651](http://www.uniprot.org/uniprot/P09651) | 38691 | 9.26 |  |  | ✓ |
| gi|4504447 | heterogeneous nuclear ribonucleoprotein A2/B1 isoform A2 | HNRPA2B1 | | [P22626](http://www.uniprot.org/uniprot/P22626) | 37407 | 8.97 |  |  | ✓ |
| gi|14110414 | heterogeneous nuclear ribonucleoprotein D isoform c | HNRPD | | [Q14103](http://www.uniprot.org/uniprot/Q14103) | 38410 | 7.61 |  |  | ✓ |
| gi|4826760 | heterogeneous nuclear ribonucleoprotein F | HNRPF | | [P52597](http://www.uniprot.org/uniprot/P52597) | 45643 | 5.38 |  |  | ✓ |
| gi|14165435 | heterogeneous nuclear ribonucleoprotein K isoform b | HNRPK | | [P61978](http://www.uniprot.org/uniprot/P61978) | 50944 | 5.39 |  |  | ✓ |
| gi|52632383 | heterogeneous nuclear ribonucleoprotein L isoform a | HNRPL | | [P14866](http://www.uniprot.org/uniprot/P14866) | 64092 | 8.46 |  |  | ✓ |
| gi|5031755 | heterogeneous nuclear ribonucleoprotein R | HNRPR | | [O43390](http://www.uniprot.org/uniprot/O43390) | 70768 | 8.25 |  |  | ✓ |
| gi|4504391 | hexokinase 1 isoform HKI | HK1 | | [P19367](http://www.uniprot.org/uniprot/P19367) | 102420 | 6.36 |  |  | ✓ |
| gi|4504333 | histidine ammonia-lyase | HAL | | [P42357](http://www.uniprot.org/uniprot/P42357) | 72652 | 6.49 |  |  | ✓ |
| gi|4758112 | HLA-B associated transcript 1 | BAT1 | | [Q13838](http://www.uniprot.org/uniprot/Q13838) | 48960 | 5.44 |  |  | ✓ |
| gi|20127408 | hydroxyacyl dehydrogenase, subunit A | HADHA | | [P40939](http://www.uniprot.org/uniprot/P40939) | 78960 | 8.98 |  |  | ✓ |
| gi|27734891 | hypothetical protein LOC286464 | CXorf59 | | [Q8N9S7](http://www.uniprot.org/uniprot/Q8N9S7) | 58020 | 6.44 |  |  | ✓ |
| gi|51093863 | hypothetical protein LOC64855 | FAM129B | | [Q96TA1](http://www.uniprot.org/uniprot/Q96TA1) | 82631 | 5.81 |  |  | ✓ |
| gi|29171702 | inorganic pyrophosphatase 2 isoform 1 | PPA2 | | [Q9H2U2](http://www.uniprot.org/uniprot/Q9H2U2) | 34685 | 5.97 |  |  | ✓ |
| gi|10835023 | inositol 1,4,5-triphosphate receptor, type 1 | ITPR1 | | [Q14643](http://www.uniprot.org/uniprot/Q14643) | 313745 | 5.71 |  |  | ✓ |
| gi|4504793 | inositol 1,4,5-triphosphate receptor, type 2 | ITPR2 | | [Q14571](http://www.uniprot.org/uniprot/Q14571) | 307881 | 6.03 |  |  | ✓ |
| gi|19743815 | integrin beta 1 isoform 1B precursor | ITGB1 | | [P05556](http://www.uniprot.org/uniprot/P05556) | 86135 | 5.27 |  |  | ✓ |
| gi|19743819 | integrin beta 1 isoform 1D precursor | ITGB1 | | [P05556](http://www.uniprot.org/uniprot/P05556) | 86135 | 5.27 |  |  | ✓ |
| gi|4758606 | integrin-linked kinase | ILK | | [Q13418](http://www.uniprot.org/uniprot/Q13418) | 51386 | 8.3 |  |  | ✓ |
| gi|4504781 | inter-alpha (globulin) inhibitor H1 | ITIH1 | | [P19827](http://www.uniprot.org/uniprot/P19827) | 71370 | 6.33 |  |  | ✓ |
| gi|31542984 | inter-alpha (globulin) inhibitor H4 | ITIH4 | | [Q14624](http://www.uniprot.org/uniprot/Q14624) | 70542 | 5.92 |  |  | ✓ |
| gi|14249342 | internexin neuronal intermediate filament protein, alpha | INA | | [Q16352](http://www.uniprot.org/uniprot/Q16352) | 55357 | 5.34 |  |  | ✓ |
| gi|4506787 | IQ motif containing GTPase activating protein 1 | IQGAP1 | | [P46940](http://www.uniprot.org/uniprot/P46940) | 189003 | 6.08 |  |  | ✓ |
| gi|28178832 | isocitrate dehydrogenase 2 (NADP+), mitochondrial precursor | IDH2 | | [P48735](http://www.uniprot.org/uniprot/P48735) | 46585 | 8.32 |  |  | ✓ |
| gi|4504811 | junction plakoglobin | JUP | | [P14923](http://www.uniprot.org/uniprot/P14923) | 81693 | 5.75 | ✓ |  | ✓ |
| gi|19923142 | karyopherin beta 1 | KPNB1 | | [Q14974](http://www.uniprot.org/uniprot/Q14974) | 97108 | 4.68 |  |  | ✓ |
| gi|17318569 | keratin 1 | KRT1 | | [P04264](http://www.uniprot.org/uniprot/P04264) | 65868 | 8.15 | ✓ | ✓ | ✓ |
| gi|40354192 | keratin 10 | KRT10 | | [P13645](http://www.uniprot.org/uniprot/P13645) | 59475 | 5.13 | ✓ | ✓ | ✓ |
| gi|4557699 | keratin 12 | KRT12 | | [Q99456](http://www.uniprot.org/uniprot/Q99456) | 53478 | 4.7 |  |  | ✓ |
| gi|24234696 | keratin 13 isoform a | KRT13 | | [P13646](http://www.uniprot.org/uniprot/P13646) | 49555 | 4.91 |  |  | ✓ |
| gi|4504911 | keratin 13 isoform b | KRT13 | | [P13646](http://www.uniprot.org/uniprot/P13646) | 49555 | 4.91 |  |  | ✓ |
| gi|15431310 | keratin 14 | KRT14 | | [P02533](http://www.uniprot.org/uniprot/P02533) | 51398 | 5.09 | ✓ | ✓ | ✓ |
| gi|24430190 | keratin 15 | KRT15 | | [P19012](http://www.uniprot.org/uniprot/P19012) | 49167 | 4.71 | ✓ | ✓ | ✓ |
| gi|24430192 | keratin 16 | KRT16 | | [P08779](http://www.uniprot.org/uniprot/P08779) | 51105 | 4.98 | ✓ |  | ✓ |
| gi|4557701 | keratin 17 | KRT17 | | [Q04695](http://www.uniprot.org/uniprot/Q04695) | 47945 | 4.97 | ✓ | ✓ | ✓ |
| gi|4557888 | keratin 18 | KRT18 | | [P05783](http://www.uniprot.org/uniprot/P05783) | 47897 | 5.34 |  |  | ✓ |
| gi|24234699 | keratin 19 | KRT19 | | [P08727](http://www.uniprot.org/uniprot/P08727) | 44065 | 5.05 | ✓ |  | ✓ |
| gi|45597458 | keratin 1B | KRT77 | | [Q7Z794](http://www.uniprot.org/uniprot/Q7Z794) | 61650 | 5.63 |  | ✓ | ✓ |
| gi|27894337 | keratin 20 | KRT20 | | [P35900](http://www.uniprot.org/uniprot/P35900) | 48457 | 5.52 |  |  | ✓ |
| gi|9506669 | keratin 24 | KRT24 | | [Q2M2I5](http://www.uniprot.org/uniprot/Q2M2I5) | 55053 | 4.89 |  |  | ✓ |
| gi|31559829 | keratin 25A | KRT25 | | [Q7Z3Z0](http://www.uniprot.org/uniprot/Q7Z3Z0) | 49287 | 5 |  |  | ✓ |
| gi|31559819 | keratin 25C | KRT27 | | [Q7Z3Y8](http://www.uniprot.org/uniprot/Q7Z3Y8) | 49793 | 5.06 |  |  | ✓ |
| gi|31559823 | keratin 25D | KRT28 | | [Q7Z3Y7](http://www.uniprot.org/uniprot/Q7Z3Y7) | 50564 | 5.33 |  |  | ✓ |
| gi|47132620 | keratin 2a | KRT2 | | [P35908](http://www.uniprot.org/uniprot/P35908) | 65393 | 8.07 | ✓ | ✓ | ✓ |
| gi|17318572 | keratin 3 | KRT3 | | [P12035](http://www.uniprot.org/uniprot/P12035) | 64465 | 6.12 | ✓ |  | ✓ |
| gi|17318574 | keratin 4 | KRT4 | | [P19013](http://www.uniprot.org/uniprot/P19013) | 57250 | 6.25 | ✓ |  | ✓ |
| gi|4557890 | keratin 5 | KRT5 | | [P13647](http://www.uniprot.org/uniprot/P13647) | 62340 | 7.58 | ✓ | ✓ | ✓ |
| gi|27597104 | keratin 5b | KRT78 | | [Q8N1N4](http://www.uniprot.org/uniprot/Q8N1N4) | 56830 | 5.79 |  |  | ✓ |
| gi|15618995 | keratin 6 irs | KRT71 | | [Q3SY84](http://www.uniprot.org/uniprot/Q3SY84) | 57214 | 6.27 |  |  | ✓ |
| gi|28173564 | keratin 6 irs3 | KRT73 | | [Q86Y46](http://www.uniprot.org/uniprot/Q86Y46) | 58887 | 6.93 | ✓ |  | ✓ |
| gi|28173552 | keratin 6 irs4 | KRT74 | | [Q7RTS7](http://www.uniprot.org/uniprot/Q7RTS7) | 57830 | 7.59 | ✓ |  | ✓ |
| gi|27465517 | keratin 6 isoform K6e | KRT6C | | [P48668](http://www.uniprot.org/uniprot/P48668) | 59857 | 8.14 |  |  | ✓ |
| gi|5031839 | keratin 6A | KRT6A | | [P02538](http://www.uniprot.org/uniprot/P02538) | 59877 | 8.14 | ✓ | ✓ | ✓ |
| gi|5031841 | keratin 6B | KRT6B | | [P04259](http://www.uniprot.org/uniprot/P04259) | 59899 | 8.14 |  |  | ✓ |
| gi|17505189 | keratin 6C | KRT6C | | [P48668](http://www.uniprot.org/uniprot/P48668) | 59857 | 8.14 |  |  | ✓ |
| gi|32567786 | keratin 6L | KRT6L | | [Q5XKE5](http://www.uniprot.org/uniprot/Q5XKE5) | 57774 | 6.75 | ✓ | ✓ | ✓ |
| gi|67782365 | keratin 7 | KRT7 | | [P08729](http://www.uniprot.org/uniprot/P08729) | 51255 | 5.5 |  |  | ✓ |
| gi|4504919 | keratin 8 | KRT8 | | [P05787](http://www.uniprot.org/uniprot/P05787) | 53540 | 5.52 | ✓ |  | ✓ |
| gi|125628636 | keratin 80 isoform a | KRT80 | | [Q6KB66](http://www.uniprot.org/uniprot/Q6KB66) | 50494 | 5.58 |  |  | ✓ |
| gi|55956899 | keratin 9 | KRT9 | | [P35527](http://www.uniprot.org/uniprot/P35527) | 62027 | 5.14 |  | ✓ | ✓ |
| gi|28372503 | keratin protein K6irs | KRT72 | | [Q14CN4](http://www.uniprot.org/uniprot/Q14CN4) | 55842 | 6.53 |  |  | ✓ |
| gi|15431316 | keratin, hair, basic, 4 | KRT84 | | [Q9NSB2](http://www.uniprot.org/uniprot/Q9NSB2) | 64855 | 8.01 | ✓ |  | ✓ |
| gi|4504935 | keratin, hair, basic, 5 | KRT85 | | [P78386](http://www.uniprot.org/uniprot/P78386) | 55766 | 6.27 | ✓ |  |  |
| gi|46409408 | keratinocyte differentiation-associated protein | UNQ467 | | [P60985](http://www.uniprot.org/uniprot/P60985) | 8764 | 5.77 |  |  | ✓ |
| gi|13699826 | kinesin family member 25 isoform 1 | KIF25 | | [Q9UIL4](http://www.uniprot.org/uniprot/Q9UIL4) | 40661 | 8.61 |  |  | ✓ |
| gi|5174457 | kinetochore associated 2 | NDC80 | | [O14777](http://www.uniprot.org/uniprot/O14777) | 73867 | 5.48 |  |  | ✓ |
| gi|4504893 | kininogen 1 | KNG1 | | [P01042](http://www.uniprot.org/uniprot/P01042) | 69853 | 6.23 |  |  | ✓ |
| gi|4885387 | L-3-hydroxyacyl-Coenzyme A dehydrogenase, short chain | HADH | | [Q16836](http://www.uniprot.org/uniprot/Q16836) | 32802 | 8.38 |  |  | ✓ |
| gi|5031857 | lactate dehydrogenase A | LDHA | | [P00338](http://www.uniprot.org/uniprot/P00338) | 36534 | 8.46 | ✓ |  | ✓ |
| gi|4557032 | lactate dehydrogenase B | LDHB | | [P07195](http://www.uniprot.org/uniprot/P07195) | 36484 | 5.72 |  | ✓ | ✓ |
| gi|27436946 | lamin A/C isoform 1 precursor | LMNA | | [P02545](http://www.uniprot.org/uniprot/P02545) | 74095 | 6.57 |  |  | ✓ |
| gi|5031875 | lamin A/C isoform 2 | LMNA | | [P02545](http://www.uniprot.org/uniprot/P02545) | 74095 | 6.57 |  | ✓ | ✓ |
| gi|27436951 | lamin B2 | LMNB2 | | [Q03252](http://www.uniprot.org/uniprot/Q03252) | 67647 | 5.29 |  |  | ✓ |
| gi|38045910 | laminin alpha 3 subunit isoform 1 | LAMA3 | | [Q16787](http://www.uniprot.org/uniprot/Q16787) | 362901 | 6.88 |  |  | ✓ |
| gi|5174445 | lanthionine synthetase C-like protein 1 | PRO0132 | | [O43813](http://www.uniprot.org/uniprot/O43813) | 45254 | 7.86 |  |  | ✓ |
| gi|24308207 | leucine rich repeat containing 47 | LRRC47 | | [Q8N1G4](http://www.uniprot.org/uniprot/Q8N1G4) | 63303 | 8.56 |  |  | ✓ |
| gi|5453710 | LIM and SH3 protein 1 | LASP1 | | [Q14847](http://www.uniprot.org/uniprot/Q14847) | 29698 | 6.61 |  |  | ✓ |
| gi|5031887 | LIM domain containing preferred translocation partner in lipoma | LPP | | [Q93052](http://www.uniprot.org/uniprot/Q93052) | 65704 | 7.18 |  |  | ✓ |
| gi|34878695 | limkain b1 | LKAP | | [Q9Y4F3](http://www.uniprot.org/uniprot/Q9Y4F3) | 192576 | 8.14 |  |  | ✓ |
| gi|4504965 | L-plastin isoform 5 | LCP1 | | [P13796](http://www.uniprot.org/uniprot/P13796) | 70114 | 5.2 |  |  | ✓ |
| gi|4505047 | lumican precursor | LUM | | [P51884](http://www.uniprot.org/uniprot/P51884) | 36638 | 6.17 | ✓ |  | ✓ |
| gi|42789729 | lymphoid-restricted membrane protein | LRMP | | [Q12912](http://www.uniprot.org/uniprot/Q12912) | 62069 | 5.62 |  |  | ✓ |
| gi|20127486 | mannose 6 phosphate receptor binding protein 1 | M6PRBP1 | | [O60664](http://www.uniprot.org/uniprot/O60664) | 47018 | 5.3 |  |  | ✓ |
| gi|4503001 | mast cell carboxypeptidase A3 precursor | CPA3 | | [P15088](http://www.uniprot.org/uniprot/P15088) | 36030 | 9.49 | ✓ |  |  |
| gi|16933567 | mel transforming oncogene | RAB8A | | [P61006](http://www.uniprot.org/uniprot/P61006) | 23328 | 9.15 |  |  | ✓ |
| gi|13699868 | methylenetetrahydrofolate dehydrogenase 1 | MTHFD1 | | [P11586](http://www.uniprot.org/uniprot/P11586) | 101364 | 6.94 |  |  | ✓ |
| gi|23111005 | microfibrillar-associated protein 4 | MFAP4 | | [P55083](http://www.uniprot.org/uniprot/P55083) | 26446 | 5.21 |  |  | ✓ |
| gi|25777732 | mitochondrial aldehyde dehydrogenase 2 precursor | ALDH2 | | [P05091](http://www.uniprot.org/uniprot/P05091) | 54410 | 5.69 |  |  | ✓ |
| gi|21735621 | mitochondrial malate dehydrogenase precursor | MDH2 | | [P40926](http://www.uniprot.org/uniprot/P40926) | 32979 | 8.54 | ✓ | ✓ | ✓ |
| gi|4505257 | moesin | MSN | | [P26038](http://www.uniprot.org/uniprot/P26038) | 67647 | 6.09 |  |  | ✓ |
| gi|4885477 | myoglobin | MB | | [P02144](http://www.uniprot.org/uniprot/P02144) | 17042 | 7.29 |  | ✓ |  |
| gi|27764861 | myosin heavy chain 6 | MYH6 | | [P13533](http://www.uniprot.org/uniprot/P13533) | 223595 | 5.6 | ✓ |  |  |
| gi|5453740 | myosin regulatory light chain MRCL3 | MYL12A | | [P19105](http://www.uniprot.org/uniprot/P19105) | 19650 | 4.67 |  |  | ✓ |
| gi|29568111 | myosin regulatory light polypeptide 9 isoform a | MYL9 | | [P24844](http://www.uniprot.org/uniprot/P24844) | 19683 | 4.8 |  |  | ✓ |
| gi|7669506 | myosin, heavy polypeptide 1, skeletal muscle, adult | MYH1 | | [P12882](http://www.uniprot.org/uniprot/P12882) | 223006 | 5.59 | ✓ |  |  |
| gi|33563340 | myosin, heavy polypeptide 14 | MYH14 | | [Q7Z406](http://www.uniprot.org/uniprot/Q7Z406) | 227732 | 5.76 |  |  | ✓ |
| gi|42476190 | myosin, heavy polypeptide 2, skeletal muscle, adult | MYH2 | | [Q9UKX2](http://www.uniprot.org/uniprot/Q9UKX2) | 222906 | 5.64 | ✓ |  |  |
| gi|11024712 | myosin, heavy polypeptide 4, skeletal muscle | MYH4 | | [Q9Y623](http://www.uniprot.org/uniprot/Q9Y623) | 222932 | 5.65 | ✓ |  |  |
| gi|4557773 | myosin, heavy polypeptide 7, cardiac muscle, beta | MYH7 | | [P12883](http://www.uniprot.org/uniprot/P12883) | 222959 | 5.63 | ✓ |  |  |
| gi|4505301 | myosin, heavy polypeptide 8, skeletal muscle, perinatal | MYH8 | | [P13535](http://www.uniprot.org/uniprot/P13535) | 222625 | 5.59 | ✓ |  |  |
| gi|12667788 | myosin, heavy polypeptide 9, non-muscle | MYH9 | | [P35579](http://www.uniprot.org/uniprot/P35579) | 226261 | 5.5 |  |  | ✓ |
| gi|21361181 | Na+/K+ -ATPase alpha 1 subunit isoform a proprotein | ATP1A1 | | [P05023](http://www.uniprot.org/uniprot/P05023) | 112352 | 5.29 |  |  | ✓ |
| gi|4502271 | Na+/K+ -ATPase alpha 2 subunit proprotein | ATP1A2 | | [P50993](http://www.uniprot.org/uniprot/P50993) | 111721 | 5.43 |  |  | ✓ |
| gi|37577153 | Na+/K+ -ATPase alpha 4 subunit isoform 1 | ATP1A4 | | [Q13733](http://www.uniprot.org/uniprot/Q13733) | 114093 | 6.23 |  |  | ✓ |
| gi|8393516 | NAD(P) dependent steroid dehydrogenase-like | NSDHL | | [Q15738](http://www.uniprot.org/uniprot/Q15738) | 41874 | 8.16 |  | ✓ |  |
| gi|4758768 | NADH dehydrogenase (ubiquinone) 1 alpha subcomplex, 10, 42kDa precursor | NDUFA10 | | [O95299](http://www.uniprot.org/uniprot/O95299) | 37124 | 6.87 |  |  | ✓ |
| gi|33519475 | NADH dehydrogenase (ubiquinone) Fe-S protein 1, 75kDa precursor | NDUFS1 | | [P28331](http://www.uniprot.org/uniprot/P28331) | 76926 | 5.42 |  |  | ✓ |
| gi|20149568 | NADH dehydrogenase (ubiquinone) flavoprotein 1, 51kDa | NDUFV1 | | [P49821](http://www.uniprot.org/uniprot/P49821) | 48474 | 7.53 |  |  | ✓ |
| gi|5453760 | neural precursor cell expressed, developmentally down-regulated 8 | NEDD8 | | [Q15843](http://www.uniprot.org/uniprot/Q15843) | 8555 | 6.58 |  |  | ✓ |
| gi|4885513 | neurofilament 3 (150kDa medium) | NEFM | | [Q4QRK6](http://www.uniprot.org/uniprot/Q4QRK6) | 102415 | 4.9 |  |  | ✓ |
| gi|32483416 | neurofilament, heavy polypeptide 200kDa | NEFH | | [P12036](http://www.uniprot.org/uniprot/P12036) | 112411 | 5.99 |  |  | ✓ |
| gi|5453762 | neurofilament, light polypeptide 68kDa | NEFL | | [P07196](http://www.uniprot.org/uniprot/P07196) | 61348 | 4.64 |  |  | ✓ |
| gi|4505409 | non-metastatic cells 2, protein (NM23B) expressed in | NME2 | | [P22392](http://www.uniprot.org/uniprot/P22392) | 17287 | 8.52 |  | ✓ | ✓ |
| gi|56699488 | nuclear receptor co-repressor 2 | NCOR2 | | [Q9Y618](http://www.uniprot.org/uniprot/Q9Y618) | 274635 | 7.21 |  |  | ✓ |
| gi|20070228 | nucleobindin 1 | NUCB1 | | [Q02818](http://www.uniprot.org/uniprot/Q02818) | 51115 | 5.09 |  |  | ✓ |
| gi|52317170 | olfactory receptor, family 1, subfamily L, member 6 | OR1L6 | | [Q8NGR2](http://www.uniprot.org/uniprot/Q8NGR2) | 39489 | 9.6 |  |  | ✓ |
| gi|9257232 | orosomucoid 1 precursor | ORM1 | | [P02763](http://www.uniprot.org/uniprot/P02763) | 21547 | 5 |  | ✓ | ✓ |
| gi|4505529 | orosomucoid 2 | ORM2 | | [P19652](http://www.uniprot.org/uniprot/P19652) | 21638 | 5.12 |  |  | ✓ |
| gi|7661704 | osteoglycin preproprotein | OGN | | [P20774](http://www.uniprot.org/uniprot/P20774) | 31715 | 5.22 | ✓ |  | ✓ |
| gi|46094014 | peptide chain release factor 3 | GSPT2 | | [Q8IYD1](http://www.uniprot.org/uniprot/Q8IYD1) | 68840 | 5.31 | ✓ |  |  |
| gi|10863927 | peptidylprolyl isomerase A isoform 1 | PPIA | | [P62937](http://www.uniprot.org/uniprot/P62937) | 17870 | 7.82 | ✓ | ✓ | ✓ |
| gi|56847632 | peptidylprolyl isomerase A-like | PPIA | | [A8K220](http://www.uniprot.org/uniprot/A8K220) | 18001 | 7.68 |  |  | ✓ |
| gi|4758950 | peptidylprolyl isomerase B precursor | PPIB | | [P23284](http://www.uniprot.org/uniprot/P23284) | 20277 | 9.25 |  |  | ✓ |
| gi|5453834 | periostin, osteoblast specific factor | POSTN | | [Q15063](http://www.uniprot.org/uniprot/Q15063) | 90905 | 7.35 |  |  | ✓ |
| gi|21264345 | peripherin | PRPH | | [P41219](http://www.uniprot.org/uniprot/P41219) | 53618 | 5.37 |  |  | ✓ |
| gi|45439327 | periplakin | PPL | | [O60437](http://www.uniprot.org/uniprot/O60437) | 204554 | 5.46 |  |  | ✓ |
| gi|4505591 | peroxiredoxin 1 | PRDX1 | | [Q06830](http://www.uniprot.org/uniprot/Q06830) | 22096 | 8.27 |  | ✓ | ✓ |
| gi|32189392 | peroxiredoxin 2 isoform a | PRDX2 | | [P32119](http://www.uniprot.org/uniprot/P32119) | 21747 | 5.67 |  | ✓ | ✓ |
| gi|33188452 | peroxiredoxin 2 isoform b | PRDX2 | |  | 16036 | 6.13 | ✓ |  |  |
| gi|5802974 | peroxiredoxin 3 isoform a precursor | PRDX3 | | [P30048](http://www.uniprot.org/uniprot/P30048) | 21455 | 5.77 |  | ✓ |  |
| gi|6912238 | peroxiredoxin 5 precursor, isoform a | PRDX5 | | [P30044](http://www.uniprot.org/uniprot/P30044) | 17020 | 6.73 |  |  | ✓ |
| gi|4758638 | peroxiredoxin 6 | PRDX6 | | [P30041](http://www.uniprot.org/uniprot/P30041) | 24888 | 6.02 |  |  | ✓ |
| gi|70995211 | peroxisomal enoyl-coenzyme A hydratase-like protein | ECH1 | | [Q13011](http://www.uniprot.org/uniprot/Q13011) | 32185 | 5.99 |  |  | ✓ |
| gi|63055049 | phosphoglucomutase 2 | PGM2 | | [Q96G03](http://www.uniprot.org/uniprot/Q96G03) | 68109 | 6.29 |  |  | ✓ |
| gi|23308577 | phosphoglycerate dehydrogenase | PHGDH | | [O43175](http://www.uniprot.org/uniprot/O43175) | 56483 | 6.31 |  |  | ✓ |
| gi|4505763 | phosphoglycerate kinase 1 | PGK1 | | [P00558](http://www.uniprot.org/uniprot/P00558) | 44455 | 8.3 | ✓ | ✓ | ✓ |
| gi|4505753 | phosphoglycerate mutase 1 (brain) | hCG_2015138 | | [P18669](http://www.uniprot.org/uniprot/P18669) | 28655 | 6.75 |  |  | ✓ |
| gi|5453539 | phosphoribosylaminoimidazole carboxylase | PAICS | | [P22234](http://www.uniprot.org/uniprot/P22234) | 46918 | 7.09 |  |  | ✓ |
| gi|10863955 | phosphoserine aminotransferase isoform 2 | PSAT1 | | [Q9Y617](http://www.uniprot.org/uniprot/Q9Y617) | 40397 | 7.56 |  |  | ✓ |
| gi|53729344 | plakophilin 1 isoform 1a | PKP1 | | [Q14CA0](http://www.uniprot.org/uniprot/Q14CA0) | 80445 | 9.18 |  |  | ✓ |
| gi|53729346 | plakophilin 1 isoform 1b | PKP1 | | [Q13835](http://www.uniprot.org/uniprot/Q13835) | 82808 | 9.29 |  |  | ✓ |
| gi|6005830 | plakophilin 3 | PKP3 | | [Q9Y446](http://www.uniprot.org/uniprot/Q9Y446) | 87029 | 9.39 | ✓ |  | ✓ |
| gi|4505897 | plastin 1 | PLS1 | | [Q14651](http://www.uniprot.org/uniprot/Q14651) | 70209 | 5.28 |  |  | ✓ |
| gi|7549809 | plastin 3 | PLS3 | | [P13797](http://www.uniprot.org/uniprot/P13797) | 70766 | 5.41 |  |  | ✓ |
| gi|47607492 | plectin 1 isoform 1 | PLEC1 | | [Q15149](http://www.uniprot.org/uniprot/Q15149) | 531466 | 5.74 |  |  | ✓ |
| gi|41322923 | plectin 1 isoform 11 | PLEC1 | | [Q15149](http://www.uniprot.org/uniprot/Q15149) | 531466 | 5.74 |  |  | ✓ |
| gi|41322908 | plectin 1 isoform 3 | PLEC1 | | [Q15149](http://www.uniprot.org/uniprot/Q15149) | 531466 | 5.74 |  |  | ✓ |
| gi|41322916 | plectin 1 isoform 6 | PLEC1 | | [Q15149](http://www.uniprot.org/uniprot/Q15149) | 531466 | 5.74 |  |  | ✓ |
| gi|4505937 | polymerase (DNA directed), gamma | POLG | | [P54098](http://www.uniprot.org/uniprot/P54098) | 139473 | 6.46 |  |  | ✓ |
| gi|42734430 | polymerase I and transcript release factor | PTRF | | [Q6NZI2](http://www.uniprot.org/uniprot/Q6NZI2) | 43450 | 5.51 |  |  | ✓ |
| gi|4506243 | polypyrimidine tract-binding protein 1 isoform a | PTBP1 | | [P26599](http://www.uniprot.org/uniprot/P26599) | 57186 | 9.22 |  |  | ✓ |
| gi|89024829 | PREDICTED: hypothetical protein LOC256355 |  | |  | 36466 | 10.43 |  |  | ✓ |
| gi|89077052 | PREDICTED: hypothetical protein LOC441426 |  | |  | 16738 | 10.24 |  |  | ✓ |
| gi|51470873 | PREDICTED: hypothetical protein XP_374880 |  | |  | 26003 | 4.95 |  |  | ✓ |
| gi|89034163 | PREDICTED: hypothetical protein XP_938379 |  | |  | 30509 |  |  |  | ✓ |
| gi|89058917 | PREDICTED: similar to 40S ribosomal protein S10 | | |  | 34162 | 10.63 |  |  | ✓ |
| gi|51474392 | PREDICTED: similar to 40S ribosomal protein S16 | | |  | 34725 | 9.8 |  |  | ✓ |
| gi|89058406 | PREDICTED: similar to actin-like protein |  | |  | 49955 | 5.61 |  |  | ✓ |
| gi|89037231 | PREDICTED: similar to actin-like protein |  | |  | 50082 | 5.79 |  |  | ✓ |
| gi|89061898 | PREDICTED: similar to Aldo-keto reductase family 1 member C1 | | | | 36624 | 9.27 |  |  | ✓ |
| gi|41151578 | PREDICTED: similar to ATP-dependent DNA helicase II, 70 kDa subunit | | | | 61595 | 8.58 |  |  | ✓ |
| gi|89060335 | PREDICTED: similar to ATP-dependent DNA helicase II, 70 kDa subunit | | | | 56725 |  |  |  | ✓ |
| gi|89025412 | PREDICTED: similar to Elongation factor 1-gamma isoform 9 | | | | 38903 | 11.14 |  |  | ✓ |
| gi|89028962 | PREDICTED: similar to epiplakin 1 |  | |  | 336901 | 5.58 |  |  | ✓ |
| gi|89028315 | PREDICTED: similar to epiplakin 1 |  | |  | 277519 | 5.68 |  |  | ✓ |
| gi|88953440 | PREDICTED: similar to eukaryotic translation elongation factor 1 alpha 2 isoform 1 | | | | 28814 | 8.51 |  |  | ✓ |
| gi|89031051 | PREDICTED: similar to Glutamate dehydrogenase 1, mitochondrial precursor | | | | 12541 | 7.82 |  |  | ✓ |
| gi|89047119 | PREDICTED: similar to Heterogeneous nuclear ribonucleoprotein A1 | | | | 26463 | 9.36 |  |  | ✓ |
| gi|88953883 | PREDICTED: similar to Heterogeneous nuclear ribonucleoprotein A1 |  | | | 35525 | 8.97 |  |  | ✓ |
| gi|89037933 | PREDICTED: similar to Ig alpha-1 chain C region isoform 1 | | |  | 30679 | 6.64 |  |  | ✓ |
| gi|89037890 | PREDICTED: similar to Ig gamma-1 chain C region | | |  | 69760 | 7.22 | ✓ | ✓ | ✓ |
| gi|89061568 | PREDICTED: similar to Ig gamma-2 chain C region | | |  | 40596 | 8.66 | ✓ | ✓ | ✓ |
| gi|89061564 | PREDICTED: similar to Ig gamma-2 chain C region | | |  | 46100 | 8.61 |  |  | ✓ |
| gi|89062143 | PREDICTED: similar to Ig gamma-4 chain C region | | |  | 52047 | 5.76 |  |  | ✓ |
| gi|89037912 | PREDICTED: similar to Ig heavy chain V-III region VH26 precursor | | | | 21468 | 9.06 |  |  | ✓ |
| gi|89042551 | PREDICTED: similar to keratin 17 isoform 1 |  | |  | 20752 |  |  |  | ✓ |
| gi|89041314 | PREDICTED: similar to keratin 17 isoform 3 |  | |  | 21722 | 4.96 |  |  | ✓ |
| gi|89041273 | PREDICTED: similar to Keratin, type I cytoskeletal 16 | | | | 15873 | 5.39 |  |  | ✓ |
| gi|89035518 | PREDICTED: similar to Keratin, type II cytoskeletal 2 oral | | | | 38824 | 9.12 |  |  | ✓ |
| gi|88988823 | PREDICTED: similar to Keratin, type II cytoskeletal 8 | | | | 24179 | 5.47 |  |  | ✓ |
| gi|89028048 | PREDICTED: similar to Keratin, type II cytoskeletal 8 isoform 1 | | | | 52769 | 5.09 |  |  | ✓ |
| gi|89028052 | PREDICTED: similar to Keratin, type II cytoskeletal 8 isoform 3 | | | | 35909 | 4.97 |  |  | ✓ |
| gi|89028056 | PREDICTED: similar to Keratin, type II cytoskeletal 8 isoform 5 | | | | 51203 | 5.19 |  |  | ✓ |
| gi|89025423 | PREDICTED: similar to monoacylglycerol O-acyltransferase 2 | | | | 38157 | 10.19 |  |  | ✓ |
| gi|89064523 | PREDICTED: similar to plakophilin 1 isoform 1a |  | |  | 72433 | 9.01 |  |  | ✓ |
| gi|88953685 | PREDICTED: similar to Prostate, ovary, testis expressed protein on chromosome 2 | | | | 129549 | 5.72 |  |  | ✓ |
| gi|88953559 | PREDICTED: similar to Prostate, ovary, testis expressed protein on chromosome 2 isoform 1 | | | | 135093 | 5.83 |  |  | ✓ |
| gi|88953571 | PREDICTED: similar to Prostate, ovary, testis expressed protein on chromosome 2 isoform 2 | | | | 135075 | 5.83 |  |  | ✓ |
| gi|88954948 | PREDICTED: similar to ribosomal protein S2 isoform 1 | | |  | 35248 | 10.09 |  |  | ✓ |
| gi|51458539 | PREDICTED: similar to ribosomal protein S2 isoform 1 | | |  | 33572 | 9.97 |  |  | ✓ |
| gi|37545481 | PREDICTED: similar to telomeric repeat binding factor 1 isoform 2 | | | | 52827 | 8.46 |  |  | ✓ |
| gi|89024839 | PREDICTED: similar to tropomyosin 3 isoform 2 | | |  | 28087 | 4.47 |  |  | ✓ |
| gi|89028749 | PREDICTED: similar to tropomyosin 4 |  | |  | 28545 | 5.08 |  |  | ✓ |
| gi|51467148 | PREDICTED: similar to tropomyosin 4 |  | |  | 28605 |  |  |  | ✓ |
| gi|88981263 | PREDICTED: similar to Tubulin beta-4q chain isoform 2 | | |  | 48500 | 5.68 |  |  | ✓ |
| gi|89030116 | PREDICTED: similar to tubulin, beta 8 isoform 1 | | |  | 56309 | 5.05 |  |  | ✓ |
| gi|89030114 | PREDICTED: similar to tubulin, beta 8 isoform 2 | | |  | 44133 | 4.77 |  |  | ✓ |
| gi|88943644 | PREDICTED: similar to ubiquitin and ribosomal protein S27a precursor | | | | 21880 | 9.53 |  |  | ✓ |
| gi|24475851 | premature ovarian failure, 1B | POF1B | | [Q8WVV4](http://www.uniprot.org/uniprot/Q8WVV4) | 68653 | 5.91 |  |  | ✓ |
| gi|4502951 | procollagen, type III, alpha 1 | COL3A1 | | [P02461](http://www.uniprot.org/uniprot/P02461) | 95230 | 9.36 |  |  | ✓ |
| gi|4505773 | prohibitin | PHB | | [P35232](http://www.uniprot.org/uniprot/P35232) | 29786 | 5.57 |  |  | ✓ |
| gi|4506041 | proline arginine-rich end leucine-rich repeat protein precursor | PRELP | | [P51888](http://www.uniprot.org/uniprot/P51888) | 41620 | 9.45 |  |  | ✓ |
| gi|20070125 | prolyl 4-hydroxylase, beta subunit | P4HB | | [P07237](http://www.uniprot.org/uniprot/P07237) | 55260 | 4.69 |  | ✓ | ✓ |
| gi|4505903 | promyelocytic leukemia protein isoform 6 | PML | | [P29590](http://www.uniprot.org/uniprot/P29590) | 97489 | 5.88 |  |  | ✓ |
| gi|18104967 | prostaglandin-endoperoxide synthase 1 isoform 1 precursor | PTGS1 | | [P23219](http://www.uniprot.org/uniprot/P23219) | 65953 | 6.69 |  |  | ✓ |
| gi|4505621 | prostatic binding protein | PEBP1 | | [P30086](http://www.uniprot.org/uniprot/P30086) | 20913 | 7.43 |  |  | ✓ |
| gi|4506145 | protease, serine, 1 preproprotein | PRSS1 | | [P07477](http://www.uniprot.org/uniprot/P07477) | 24099 | 7.64 |  |  | ✓ |
| gi|7661914 | proteasome (prosome, macropain) 26S subunit, non-ATPase, 6 | PSMD6 | | [Q15008](http://www.uniprot.org/uniprot/Q15008) | 45371 | 5.45 |  |  | ✓ |
| gi|30410792 | proteasome activator subunit 2 | PSME2 | | [Q9UL46](http://www.uniprot.org/uniprot/Q9UL46) | 27213 | 5.44 |  |  | ✓ |
| gi|22538465 | proteasome beta 3 subunit | PSMB3 | | [P49720](http://www.uniprot.org/uniprot/P49720) | 22802 | 6.13 |  |  | ✓ |
| gi|6005826 | protein kinase C and casein kinase substrate in neurons 2 | PACSIN2 | | [Q9UNF0](http://www.uniprot.org/uniprot/Q9UNF0) | 55704 | 5.08 |  |  | ✓ |
| gi|13654237 | protein kinase, DNA-activated, catalytic polypeptide | PRKDC | | [P78527](http://www.uniprot.org/uniprot/P78527) | 468788 | 6.75 |  |  | ✓ |
| gi|4506005 | protein phosphatase 1, catalytic subunit, beta isoform 1 | PPP1CB | | [P62140](http://www.uniprot.org/uniprot/P62140) | 37032 | 5.85 |  |  | ✓ |
| gi|4506007 | protein phosphatase 1, catalytic subunit, gamma isoform | PPP1CC | | [P36873](http://www.uniprot.org/uniprot/P36873) | 36829 | 6.14 |  |  | ✓ |
| gi|29557855 | protein phosphatase 1A isoform 2 | PPM1A | | [P35813](http://www.uniprot.org/uniprot/P35813) | 42421 | 5.19 |  |  | ✓ |
| gi|4506017 | protein phosphatase 2, catalytic subunit, alpha isoform | PPP2CA | | [P67775](http://www.uniprot.org/uniprot/P67775) | 35571 | 5.3 |  |  | ✓ |
| gi|4885539 | protein-L-isoaspartate (D-aspartate) O-methyltransferase | PCMT1 | | [P22061](http://www.uniprot.org/uniprot/P22061) | 24504 | 6.78 |  |  | ✓ |
| gi|14195605 | protocadherin beta 16 precursor | PCDHB16 | | [Q9NRJ7](http://www.uniprot.org/uniprot/Q9NRJ7) | 81704 | 4.91 |  |  | ✓ |
| gi|11128037 | protocadherin gamma subfamily B, 1 isoform 1 precursor | PCDHGB1 | | [Q9Y5G3](http://www.uniprot.org/uniprot/Q9Y5G3) | 97113 | 4.89 |  |  | ✓ |
| gi|5454002 | putative c-Myc-responsive isoform 1 | C6orf108 | | [O43598](http://www.uniprot.org/uniprot/O43598) | 19097 | 4.97 |  |  | ✓ |
| gi|33286418 | pyruvate kinase 3 isoform 1 | PKM2 | | [P14618](http://www.uniprot.org/uniprot/P14618) | 57769 | 7.95 | ✓ |  | ✓ |
| gi|33286420 | pyruvate kinase 3 isoform 2 | PKM2 | | [P14618](http://www.uniprot.org/uniprot/P14618) | 57769 | 7.95 |  |  | ✓ |
| gi|10835121 | pyruvate kinase, liver and RBC isoform 1 | PKLR | | [P30613](http://www.uniprot.org/uniprot/P30613) | 61791 | 7.65 |  |  | ✓ |
| gi|4506359 | quinoid dihydropteridine reductase | QDPR | | [P09417](http://www.uniprot.org/uniprot/P09417) | 25642 | 7.1 |  |  | ✓ |
| gi|13569962 | RAB1B, member RAS oncogene family | RAB1B | | [Q9H0U4](http://www.uniprot.org/uniprot/Q9H0U4) | 22157 | 5.55 |  |  | ✓ |
| gi|7661922 | RAB21, member RAS oncogene family | RAB21 | | [Q9UL25](http://www.uniprot.org/uniprot/Q9UL25) | 23970 | 8.16 |  |  | ✓ |
| gi|4506371 | RAB5B, member RAS oncogene family | RAB5B | | [P61020](http://www.uniprot.org/uniprot/P61020) | 23692 | 8.29 |  |  | ✓ |
| gi|51036601 | RAB6B, member RAS oncogene family | RAB6B | | [Q9NRW1](http://www.uniprot.org/uniprot/Q9NRW1) | 23447 | 5.41 |  |  | ✓ |
| gi|9845509 | ras-related C3 botulinum toxin substrate 1 isoform Rac1b | RAC1 | | [P63000](http://www.uniprot.org/uniprot/P63000) | 21097 | 8.77 |  |  | ✓ |
| gi|33695095 | ras-related GTP-binding protein RAB10 | RAB10 | | [P61026](http://www.uniprot.org/uniprot/P61026) | 22527 | 8.58 |  |  | ✓ |
| gi|27883866 | resistance to inhibitors of cholinesterase 8 homolog A | RIC8A | | [Q9NPQ8](http://www.uniprot.org/uniprot/Q9NPQ8) | 59574 | 5.2 |  |  | ✓ |
| gi|4757768 | Rho GDP dissociation inhibitor (GDI) alpha | ARHGDIA | | [P52565](http://www.uniprot.org/uniprot/P52565) | 23062 | 5.03 |  |  | ✓ |
| gi|21361547 | ribonuclease/angiogenin inhibitor | RNH1 | | [P13489](http://www.uniprot.org/uniprot/P13489) | 49810 | 4.71 |  |  | ✓ |
| gi|35493916 | ribophorin II precursor | RPN2 | | [P04844](http://www.uniprot.org/uniprot/P04844) | 67013 | 5.44 |  |  | ✓ |
| gi|4506669 | ribosomal protein P1 isoform 1 | RPLP1 | | [P05386](http://www.uniprot.org/uniprot/P05386) | 11507 | 4.26 |  |  | ✓ |
| gi|4506671 | ribosomal protein P2 | RPLP2 | | [P05387](http://www.uniprot.org/uniprot/P05387) | 11658 | 4.42 |  |  | ✓ |
| gi|4506679 | ribosomal protein S10 | RPS10 | | [P46783](http://www.uniprot.org/uniprot/P46783) | 18886 | 10.15 |  |  | ✓ |
| gi|14277700 | ribosomal protein S12 | RPS12 | | [P25398](http://www.uniprot.org/uniprot/P25398) | 14374 | 7.01 |  |  | ✓ |
| gi|4506685 | ribosomal protein S13 | RPS13 | | [P62277](http://www.uniprot.org/uniprot/P62277) | 17081 | 10.53 |  |  | ✓ |
| gi|4506691 | ribosomal protein S16 | RPS16 | | [P62249](http://www.uniprot.org/uniprot/P62249) | 16304 | 10.21 |  |  | ✓ |
| gi|15055539 | ribosomal protein S2 | RPS2 | | [P15880](http://www.uniprot.org/uniprot/P15880) | 31305 | 10.25 |  |  | ✓ |
| gi|4506697 | ribosomal protein S20 | RPS20 | | [P60866](http://www.uniprot.org/uniprot/P60866) | 13233 | 9.95 |  |  | ✓ |
| gi|13904870 | ribosomal protein S5 | RPS5 | | [P46782](http://www.uniprot.org/uniprot/P46782) | 22862 | 9.73 |  |  | ✓ |
| gi|9845502 | ribosomal protein SA | RPSA | | [P08865](http://www.uniprot.org/uniprot/P08865) | 32702 | 4.79 |  |  | ✓ |
| gi|10863871 | ryanodine receptor 1 (skeletal) | RYR1 | | [P21817](http://www.uniprot.org/uniprot/P21817) | 564815 | 5.18 |  |  | ✓ |
| gi|5032057 | S100 calcium binding protein A11 (calgizzarin) | S100A11 | | [P31949](http://www.uniprot.org/uniprot/P31949) | 11733 | 6.56 |  |  | ✓ |
| gi|10190712 | S100 calcium binding protein A14 | S100A14 | | [Q9HCY8](http://www.uniprot.org/uniprot/Q9HCY8) | 11655 | 5.16 |  |  | ✓ |
| gi|17933772 | S100 calcium binding protein A16 | S100A16 | | [Q96FQ6](http://www.uniprot.org/uniprot/Q96FQ6) | 11794 | 6.28 |  |  | ✓ |
| gi|28827815 | S100 calcium binding protein A7-like 1 | S100A7A | | [Q86SG5](http://www.uniprot.org/uniprot/Q86SG5) | 11167 | 6.87 |  |  | ✓ |
| gi|4506761 | S100 calcium-binding protein A10 | S100A10 | | [P60903](http://www.uniprot.org/uniprot/P60903) | 11064 | 7.3 |  |  | ✓ |
| gi|4506765 | S100 calcium-binding protein A4 | S100A4 | | [P26447](http://www.uniprot.org/uniprot/P26447) | 11590 | 5.88 |  |  | ✓ |
| gi|4506769 | S100 calcium-binding protein A7 | S100A7 | | [P31151](http://www.uniprot.org/uniprot/P31151) | 11333 | 6.26 |  | ✓ | ✓ |
| gi|21614544 | S100 calcium-binding protein A8 | S100A8 | | [P05109](http://www.uniprot.org/uniprot/P05109) | 10828 | 6.51 | ✓ |  |  |
| gi|4506773 | S100 calcium-binding protein A9 | S100A9 | | [P06702](http://www.uniprot.org/uniprot/P06702) | 13234 | 5.71 |  | ✓ | ✓ |
| gi|9951915 | S-adenosylhomocysteine hydrolase | AHCY | | [P23526](http://www.uniprot.org/uniprot/P23526) | 47554 | 5.92 |  | ✓ |  |
| gi|14591924 | Sec23 (S. cerevisiae) homolog B | SEC23B | | [Q15437](http://www.uniprot.org/uniprot/Q15437) | 86424 | 6.43 |  |  | ✓ |
| gi|7019415 | Sec61 alpha 1 subunit | SEC61A1 | | [P61619](http://www.uniprot.org/uniprot/P61619) | 52099 | 8.33 |  |  | ✓ |
| gi|16306550 | selenium binding protein 1 | SELENBP1 | | [Q13228](http://www.uniprot.org/uniprot/Q13228) | 52358 | 5.93 |  |  | ✓ |
| gi|4758158 | septin 2 | SEP2 | | [Q15019](http://www.uniprot.org/uniprot/Q15019) | 41461 | 6.15 |  |  | ✓ |
| gi|50363217 | serine (or cysteine) proteinase inhibitor, clade A, member 1 | SERPINA1 | | [P01009](http://www.uniprot.org/uniprot/P01009) | 44297 | 5.37 | ✓ | ✓ | ✓ |
| gi|17998551 | serine (or cysteine) proteinase inhibitor, clade B, member 12 | SERPINB12 | | [Q96P63](http://www.uniprot.org/uniprot/Q96P63) | 46247 | 5.36 |  |  | ✓ |
| gi|5902072 | serine (or cysteine) proteinase inhibitor, clade B, member 3 | SERPINB3 | | [P29508](http://www.uniprot.org/uniprot/P29508) | 44537 | 6.35 |  |  | ✓ |
| gi|4505789 | serine (or cysteine) proteinase inhibitor, clade B, member 5 | SERPINB5 | | [P36952](http://www.uniprot.org/uniprot/P36952) | 42073 | 5.72 |  | ✓ | ✓ |
| gi|41152086 | serine (or cysteine) proteinase inhibitor, clade B, member 6 | SERPINB6 | | [P35237](http://www.uniprot.org/uniprot/P35237) | 42594 | 5.18 |  |  | ✓ |
| gi|38504669 | serine (or cysteine) proteinase inhibitor, clade B, member 8 isoform a | SERPINB8 | | [P50452](http://www.uniprot.org/uniprot/P50452) | 42739 | 5.41 |  |  | ✓ |
| gi|4502261 | serine (or cysteine) proteinase inhibitor, clade C, member 1 | SERPINC1 | | [P01008](http://www.uniprot.org/uniprot/P01008) | 49008 | 5.95 |  |  | ✓ |
| gi|39725934 | serine (or cysteine) proteinase inhibitor, clade F, member 1 | SERPINF1 | | [P36955](http://www.uniprot.org/uniprot/P36955) | 44390 | 5.9 |  |  | ✓ |
| gi|32454741 | serine (or cysteine) proteinase inhibitor, clade H, member 1 precursor | SERPINH1 | | [P50454](http://www.uniprot.org/uniprot/P50454) | 44495 | 8.81 |  |  | ✓ |
| gi|50659080 | serpin peptidase inhibitor, clade A, member 3 precursor | SERPINA3 | | [P01011](http://www.uniprot.org/uniprot/P01011) | 45237 | 5.32 |  |  | ✓ |
| gi|66346679 | SERPINE1 mRNA binding protein 1 isoform 1 | SERBP1 | | [Q8NC51](http://www.uniprot.org/uniprot/Q8NC51) | 44807 | 8.72 |  |  | ✓ |
| gi|4502133 | serum amyloid P component precursor | APCS | | [P02743](http://www.uniprot.org/uniprot/P02743) | 23244 | 6.12 | ✓ | ✓ |  |
| gi|18699722 | shroom family member 3 | SHROOM3 | | [Q8TF72](http://www.uniprot.org/uniprot/Q8TF72) | 216528 | 7.75 |  |  | ✓ |
| gi|4557241 | skeletal muscle specific actinin, alpha 3 | ACTN3 | | [Q08043](http://www.uniprot.org/uniprot/Q08043) | 103176 | 5.37 |  |  | ✓ |
| gi|22758146 | Skin ASpartic Protease | ASPRV1 | | [Q53RT3](http://www.uniprot.org/uniprot/Q53RT3) | 14846 | 5.2 |  |  | ✓ |
| gi|4507125 | small nuclear ribonucleoprotein polypeptide B/B~ isoform B | SNRPB | | [P14678](http://www.uniprot.org/uniprot/P14678) | 24594 | 11.2 |  |  | ✓ |
| gi|4507129 | small nuclear ribonucleoprotein polypeptide E | SNRPE | | [P62304](http://www.uniprot.org/uniprot/P62304) | 10797 | 9.46 |  |  | ✓ |
| gi|17986258 | smooth muscle and non-muscle myosin alkali light chain isoform 1 | MYL6 | | [P60660](http://www.uniprot.org/uniprot/P60660) | 16788 | 4.56 |  |  | ✓ |
| gi|17986264 | smooth muscle and non-muscle myosin alkali light chain isoform 3 | | | | 19124 | 4.51 |  |  | ✓ |
| gi|13124879 | smooth muscle myosin heavy chain 11 isoform SM1 | MYH11 | | [P35749](http://www.uniprot.org/uniprot/P35749) | 227199 | 5.42 |  |  | ✓ |
| gi|13124875 | smooth muscle myosin heavy chain 11 isoform SM2 | MYH11 | | [Q3MIV8](http://www.uniprot.org/uniprot/Q3MIV8) | 223439 | 5.44 |  |  | ✓ |
| gi|55749577 | solute carrier family 25, member 4 | SLC25A6 | | [P12235](http://www.uniprot.org/uniprot/P12235) | 32912 | 9.78 |  |  | ✓ |
| gi|4505775 | solute carrier family 25 member 3 isoform b precursor | SLC25A3 | | [Q00325](http://www.uniprot.org/uniprot/Q00325) | 34873 | 9.29 |  |  | ✓ |
| gi|4502099 | solute carrier family 25, member 5 | SLC25A5 | | [P05141](http://www.uniprot.org/uniprot/P05141) | 32743 | 9.76 |  |  | ✓ |
| gi|27764863 | solute carrier family 25, member A6 | SLC25A6 | | [P12236](http://www.uniprot.org/uniprot/P12236) | 32714 | 9.76 |  |  | ✓ |
| gi|4507021 | solute carrier family 4, anion exchanger, member 1 | SLC4A1 | | [P02730](http://www.uniprot.org/uniprot/P02730) | 101727 | 5.08 |  |  | ✓ |
| gi|10863913 | solute carrier family 8, member 1 | SLC8A1 | | [P32418](http://www.uniprot.org/uniprot/P32418) | 104478 | 4.84 |  |  | ✓ |
| gi|23111032 | sorting nexin 1 isoform b | SNX1 | | [Q13596](http://www.uniprot.org/uniprot/Q13596) | 59033 | 5.08 |  |  | ✓ |
| gi|67782319 | spectrin beta isoform b | SPTB | | [P11277](http://www.uniprot.org/uniprot/P11277) | 246185 | 5.15 |  |  | ✓ |
| gi|4507189 | spectrin, alpha, erythrocytic 1 | SPTA1 | | [Q5VYL1](http://www.uniprot.org/uniprot/Q5VYL1) | 279842 | 4.95 |  |  | ✓ |
| gi|4507191 | spectrin, alpha, non-erythrocytic 1 (alpha-fodrin) | SPTAN1 | | [Q13813](http://www.uniprot.org/uniprot/Q13813) | 284364 | 5.22 |  |  | ✓ |
| gi|4507195 | spectrin, beta, non-erythrocytic 1 isoform 1 | SPTBN1 | | [Q01082](http://www.uniprot.org/uniprot/Q01082) | 274439 | 5.39 |  |  | ✓ |
| gi|30315658 | spectrin, beta, non-erythrocytic 1 isoform 2 | SPTBN1 | | [Q01082](http://www.uniprot.org/uniprot/Q01082) | 274439 | 5.39 |  |  | ✓ |
| gi|4826998 | splicing factor proline/glutamine rich | SFPQ | | [P23246](http://www.uniprot.org/uniprot/P23246) | 76102 | 9.45 |  |  | ✓ |
| gi|5454052 | stratifin | SFN | | [P31947](http://www.uniprot.org/uniprot/P31947) | 27757 | 4.68 | ✓ | ✓ | ✓ |
| gi|4759080 | succinate dehydrogenase complex, subunit A, flavoprotein precursor | SDHA | | [P31040](http://www.uniprot.org/uniprot/P31040) | 67969 | 6.25 |  |  | ✓ |
| gi|4507149 | superoxide dismutase 1, soluble | SOD1 | | [P00441](http://www.uniprot.org/uniprot/P00441) | 15795 | 5.7 |  |  | ✓ |
| gi|4507151 | superoxide dismutase 3, extracellular | SOD3 | | [P08294](http://www.uniprot.org/uniprot/P08294) | 24118 | 6.32 |  |  | ✓ |
| gi|38348366 | suprabasin | SBSN | | [Q6UWP8](http://www.uniprot.org/uniprot/Q6UWP8) | 22799 | 6.4 |  |  | ✓ |
| gi|19557691 | surfeit 4 | SURF4 | | [O15260](http://www.uniprot.org/uniprot/O15260) | 30374 | 7.64 |  |  | ✓ |
| gi|23397427 | synaptotagmin binding, cytoplasmic RNA interacting protein | SYNCRIP | | [O60506](http://www.uniprot.org/uniprot/O60506) | 69560 | 8.68 |  |  | ✓ |
| gi|16753233 | talin 1 | TLN1 | | [Q9Y490](http://www.uniprot.org/uniprot/Q9Y490) | 269599 | 5.77 |  |  | ✓ |
| gi|57863257 | T-complex protein 1 isoform a | TCP1 | | [P17987](http://www.uniprot.org/uniprot/P17987) | 60306 | 5.8 |  |  | ✓ |
| gi|67782336 | tenascin XB isoform 1 | TNXB | | [P22105](http://www.uniprot.org/uniprot/P22105) | 461734 | 5.19 |  |  | ✓ |
| gi|50592994 | thioredoxin | TXN | | [P10599](http://www.uniprot.org/uniprot/P10599) | 11599 | 4.82 |  |  | ✓ |
| gi|21361794 | TIP120 protein | CAND1 | | [Q86VP6](http://www.uniprot.org/uniprot/Q86VP6) | 136158 | 5.52 |  |  | ✓ |
| gi|11321603 | TNF receptor-associated factor 5 | TRAF5 | | [O00463](http://www.uniprot.org/uniprot/O00463) | 64364 | 7.26 |  |  | ✓ |
| gi|7706485 | TNF receptor-associated protein 1 | TRAP1 | | [Q12931](http://www.uniprot.org/uniprot/Q12931) | 73500 | 6.13 | ✓ |  |  |
| gi|5803187 | transaldolase 1 | TALDO1 | | [P37837](http://www.uniprot.org/uniprot/P37837) | 37516 | 6.36 | ✓ | ✓ | ✓ |
| gi|4557871 | transferrin | TF | | [P02787](http://www.uniprot.org/uniprot/P02787) | 75133 | 6.7 |  |  | ✓ |
| gi|4507467 | transforming growth factor, beta-induced, 68kDa | TGFBI | | [Q15582](http://www.uniprot.org/uniprot/Q15582) | 72373 | 7.37 |  |  | ✓ |
| gi|48255905 | transgelin | TAGLN | | [Q01995](http://www.uniprot.org/uniprot/Q01995) | 22465 | 8.88 |  |  | ✓ |
| gi|4507357 | transgelin 2 | TAGLN2 | | [P37802](http://www.uniprot.org/uniprot/P37802) | 22246 | 8.45 |  |  | ✓ |
| gi|4507521 | transketolase | TKT | | [P29401](http://www.uniprot.org/uniprot/P29401) | 67835 | 7.58 |  |  | ✓ |
| gi|4507645 | triosephosphate isomerase 1 | TPI1 | | [P60174](http://www.uniprot.org/uniprot/P60174) | 26522 | 6.51 |  | ✓ | ✓ |
| gi|17402907 | tripartite motif protein TRIM29 isoform beta | TRIM29 | | [Q14134](http://www.uniprot.org/uniprot/Q14134) | 65793 | 6.73 |  |  | ✓ |
| gi|63252902 | tropomyosin 1 alpha chain isoform 2 | TPM1 | | [Q9Y427](http://www.uniprot.org/uniprot/Q9Y427) | 32658 | 4.7 |  |  | ✓ |
| gi|63252896 | tropomyosin 1 alpha chain isoform 3 | TPM1 | | [O15513](http://www.uniprot.org/uniprot/O15513) | 38239 | 4.71 |  |  | ✓ |
| gi|63252900 | tropomyosin 1 alpha chain isoform 4 | TPM1 | | [P09493](http://www.uniprot.org/uniprot/P09493) | 32689 | 4.69 |  |  | ✓ |
| gi|27597085 | tropomyosin 1 alpha chain isoform 5 | TPM1 | | [P09493](http://www.uniprot.org/uniprot/P09493) | 32689 | 4.69 |  | ✓ | ✓ |
| gi|63252906 | tropomyosin 1 alpha chain isoform 7 | TPM1 | | [O15513](http://www.uniprot.org/uniprot/O15513) | 37408 | 4.71 |  |  | ✓ |
| gi|42476296 | tropomyosin 2 (beta) isoform 1 | TPM2 | | [P07951](http://www.uniprot.org/uniprot/P07951) | 32831 | 4.66 |  | ✓ | ✓ |
| gi|47519616 | tropomyosin 2 (beta) isoform 2 | TPM2 | | [P07951](http://www.uniprot.org/uniprot/P07951) | 32831 | 4.66 |  |  | ✓ |
| gi|24119203 | tropomyosin 3 isoform 2 | TPM3 | | [P06753](http://www.uniprot.org/uniprot/P06753) | 32799 | 4.68 |  |  | ✓ |
| gi|4507651 | tropomyosin 4 | TPM4 | | [P67936](http://www.uniprot.org/uniprot/P67936) | 28373 | 4.67 |  |  | ✓ |
| gi|22748757 | truncated type I keratin KA21 | KRT222P | | [Q8N1A0](http://www.uniprot.org/uniprot/Q8N1A0) | 34137 | 5.63 |  |  | ✓ |
| gi|13775595 | tryptase alpha/beta 1 precursor | TPSB2 | | [P20231](http://www.uniprot.org/uniprot/P20231) | 27427 | 6.3 |  |  | ✓ |
| gi|34147630 | Tu translation elongation factor, mitochondrial | TUFM | | [P49411](http://www.uniprot.org/uniprot/P49411) | 45017 | 6.31 |  |  | ✓ |
| gi|14389309 | tubulin alpha 6 | TUBA1C | | [Q9BQE3](http://www.uniprot.org/uniprot/Q9BQE3) | 49863 | 4.96 | ✓ | ✓ | ✓ |
| gi|17921989 | tubulin, alpha 1 | TUBA4A | | [P68366](http://www.uniprot.org/uniprot/P68366) | 49892 | 4.95 |  |  | ✓ |
| gi|17921993 | tubulin, alpha 2 isoform 1 | TUBA3C | | [Q13748](http://www.uniprot.org/uniprot/Q13748) | 49928 | 4.98 |  |  | ✓ |
| gi|17921991 | tubulin, alpha 2 isoform 2 |  | |  | 49928 | 4.98 |  |  | ✓ |
| gi|17986283 | tubulin, alpha 3 | TUBA1A | | [Q71U36](http://www.uniprot.org/uniprot/Q71U36) | 50104 | 4.94 | ✓ |  | ✓ |
| gi|46409270 | tubulin, alpha 3e | TUBA3E | | [Q6PEY2](http://www.uniprot.org/uniprot/Q6PEY2) | 49827 | 5.01 |  |  | ✓ |
| gi|9507215 | tubulin, alpha 8 | TUBA8 | | [Q9NY65](http://www.uniprot.org/uniprot/Q9NY65) | 50062 | 4.94 |  |  | ✓ |
| gi|57013276 | tubulin, alpha, ubiquitous | TUBA1B | | [P68363](http://www.uniprot.org/uniprot/P68363) | 50120 | 4.94 |  |  | ✓ |
| gi|4507729 | tubulin, beta 2 | TUBB2A | | [Q13885](http://www.uniprot.org/uniprot/Q13885) | 49875 | 4.78 | ✓ | ✓ | ✓ |
| gi|21361322 | tubulin, beta 4 | TUBB4 | | [P04350](http://www.uniprot.org/uniprot/P04350) | 49554 | 4.78 |  |  | ✓ |
| gi|14210536 | tubulin, beta 6 | TUBB6 | | [Q9BUF5](http://www.uniprot.org/uniprot/Q9BUF5) | 49825 | 4.77 | ✓ | ✓ | ✓ |
| gi|42558279 | tubulin, beta 8 | MAP3K8 | | [Q3ZCM7](http://www.uniprot.org/uniprot/Q3ZCM7) | 49744 | 4.79 |  |  | ✓ |
| gi|29788785 | tubulin, beta polypeptide | TUBB | | [P07437](http://www.uniprot.org/uniprot/P07437) | 49639 | 4.78 |  | ✓ | ✓ |
| gi|55770868 | tubulin, beta polypeptide 4, member Q | TUBB4Q | | [Q99867](http://www.uniprot.org/uniprot/Q99867) | 48403 | 5.11 |  |  | ✓ |
| gi|5174735 | tubulin, beta, 2 | TUBB2C | | [P68371](http://www.uniprot.org/uniprot/P68371) | 49799 | 4.79 |  | ✓ | ✓ |
| gi|50592996 | tubulin, beta, 4 | TUBB3 | | [Q13509](http://www.uniprot.org/uniprot/Q13509) | 50400 | 4.83 | ✓ | ✓ | ✓ |
| gi|4507677 | tumor rejection antigen (gp96) 1 | HSP90B1 | | [P14625](http://www.uniprot.org/uniprot/P14625) | 90122 | 4.73 |  |  | ✓ |
| gi|5803225 | tyrosine 3/tryptophan 5 -monooxygenase activation protein, epsilon polypeptide | YWHAE | | [P62258](http://www.uniprot.org/uniprot/P62258) | 29155 | 4.63 |  | ✓ | ✓ |
| gi|4507951 | tyrosine 3/tryptophan 5 -monooxygenase activation protein, eta polypeptide | YWHAH | | [Q04917](http://www.uniprot.org/uniprot/Q04917) | 28070 | 4.76 |  | ✓ | ✓ |
| gi|5803227 | tyrosine 3/tryptophan 5 -monooxygenase activation protein, theta polypeptide | YWHAQ | | [P27348](http://www.uniprot.org/uniprot/P27348) | 27747 | 4.68 | ✓ | ✓ | ✓ |
| gi|4507953 | tyrosine 3/tryptophan 5 -monooxygenase activation protein, zeta polypeptide | YWHAZ | | [P63104](http://www.uniprot.org/uniprot/P63104) | 27728 | 4.73 | ✓ | ✓ | ✓ |
| gi|4507949 | tyrosine 3-monooxygenase/tryptophan 5-monooxygenase activation protein, beta | YWHAB | | [P31946](http://www.uniprot.org/uniprot/P31946) | 28065 | 4.76 | ✓ |  | ✓ |
| gi|21464101 | tyrosine 3-monooxygenase/tryptophan 5-monooxygenase activation protein, gamma polypeptide | YWHAG | | [P61981](http://www.uniprot.org/uniprot/P61981) | 28285 | 4.8 |  | ✓ | ✓ |
| gi|50592988 | ubiquinol-cytochrome c reductase core protein II | UQCRC2 | | [P22695](http://www.uniprot.org/uniprot/P22695) | 46755 | 7.74 |  |  | ✓ |
| gi|4507761 | ubiquitin and ribosomal protein L40 precursor | UBA52 | | [Q3MIH3](http://www.uniprot.org/uniprot/Q3MIH3) | 14719 | 9.87 |  |  | ✓ |
| gi|4506713 | ubiquitin and ribosomal protein S27a precursor | UBB | | [P62979](http://www.uniprot.org/uniprot/P62979) | 9412 | 9.86 |  |  | ✓ |
| gi|67191208 | ubiquitin C | UBC | | [P62988](http://www.uniprot.org/uniprot/P62988) | 8560 | 6.56 |  |  | ✓ |
| gi|4507855 | Ubiquitin isopeptidase T | USP5 | | [P45974](http://www.uniprot.org/uniprot/P45974) | 95725 | 4.91 |  |  | ✓ |
| gi|23510338 | ubiquitin-activating enzyme E1 | UBE1 | | [P22314](http://www.uniprot.org/uniprot/P22314) | 117774 | 5.49 |  |  | ✓ |
| gi|40255039 | ubiquitin-activating enzyme E1-like 2 | UBE1L2 | | [A0AVT1](http://www.uniprot.org/uniprot/A0AVT1) | 117895 | 5.76 |  |  | ✓ |
| gi|4506387 | UV excision repair protein RAD23 homolog B | RAD23B | | [P54727](http://www.uniprot.org/uniprot/P54727) | 43145 | 4.79 |  |  | ✓ |
| gi|17999541 | vacuolar protein sorting 35 | VPS35 | | [Q96QK1](http://www.uniprot.org/uniprot/Q96QK1) | 91649 | 5.32 |  |  | ✓ |
| gi|6005942 | valosin-containing protein | VCP | | [P55072](http://www.uniprot.org/uniprot/P55072) | 89135 | 5.14 |  |  | ✓ |
| gi|4507869 | vasodilator-stimulated phosphoprotein isoform 1 | VASP | | [P50552](http://www.uniprot.org/uniprot/P50552) | 39674 | 9.05 |  |  | ✓ |
| gi|18379349 | vesicle amine transport protein 1 | VAT1 | | [Q99536](http://www.uniprot.org/uniprot/Q99536) | 41893 | 5.88 |  |  | ✓ |
| gi|21614499 | villin 2 | VIL2 | | [P15311](http://www.uniprot.org/uniprot/P15311) | 69239 | 5.95 |  |  | ✓ |
| gi|62414289 | vimentin | VIM | | [P08670](http://www.uniprot.org/uniprot/P08670) | 53488 | 5.06 | ✓ | ✓ | ✓ |
| gi|4507877 | vinculin isoform VCL | VCL | | [P18206](http://www.uniprot.org/uniprot/P18206) | 123591 | 5.51 |  |  | ✓ |
| gi|32483410 | vitamin D-binding protein precursor | GC | | [P02774](http://www.uniprot.org/uniprot/P02774) | 51210 | 5.22 |  | ✓ | ✓ |
| gi|4507879 | voltage-dependent anion channel 1 | VDAC1 | | [P21796](http://www.uniprot.org/uniprot/P21796) | 30623 | 8.63 |  |  | ✓ |
| gi|42476281 | voltage-dependent anion channel 2 | VDAC2 | | [P45880](http://www.uniprot.org/uniprot/P45880) | 31547 | 7.5 |  |  | ✓ |
| gi|18860918 | xylulokinase homolog | XYLB | | [O75191](http://www.uniprot.org/uniprot/O75191) | 58345 | 5.55 | ✓ |  |  |
