# Supplementary material for: Proteomic Analysis of the Human Skin Proteome after In Vivo Treatment with Sodium Dodecyl Sulphate
Source: PLoS One. 2014 May 21;9(5):e97772. doi: 10.1371/journal.pone.0097772 (PMC4029809; doi:10.1371/journal.pone.0097772)

# File S1

# Figure S1. SDS PAGE of skin tissue lysates prepared in different buffers.

Five skin tissue lysates were obtained via the FastPrep method using the buffers 1-5 listed in Table S1 in File S1 (Lanes 1-5, respectively). Lane M shows the molecular weight ladder. Panels A and B show the proteins obtained in the soluble and insoluble fractions, respectively.


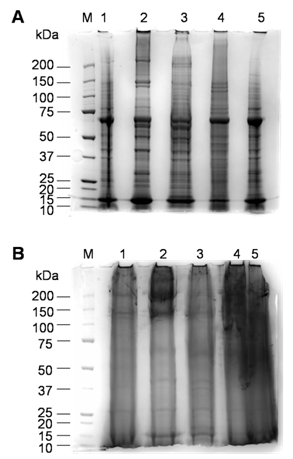


**Figure S2**. **Western Blot analysis of control and test biopsy samples from 2 volunteers showing expression of caspase-14.**

Caspase 14 was found to be expressed in both control and test biopsy samples in 2 forms, as the 29 kDa protein and also as a 10 kDa active subunit following exposure of protein lysates (resolved through a Novex 10-20% Tricine gel and transferred onto a nitrocellulose membrane) to 3 µg/ml of a mouse anti-caspase 14 antibody (Abcam, Cambridge, UK). In the SDS treated samples, expression of the 10 kDa active subunit appears to be down-regulated.


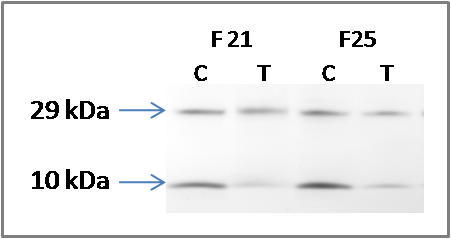
**Figure S3**. **Reproducibility of data in a representative experiment (F37F38) using 4- and 8-plex iTRAQ analysis.**

Proteins were identified in both the original iTRAQ 4-plex experiment and the two technical replicates in an iTRAQ 8-plex experiment, and the ratios between control and test samples calculated. With one exception (alpha globin), all protein ratios showed good reproducibility.


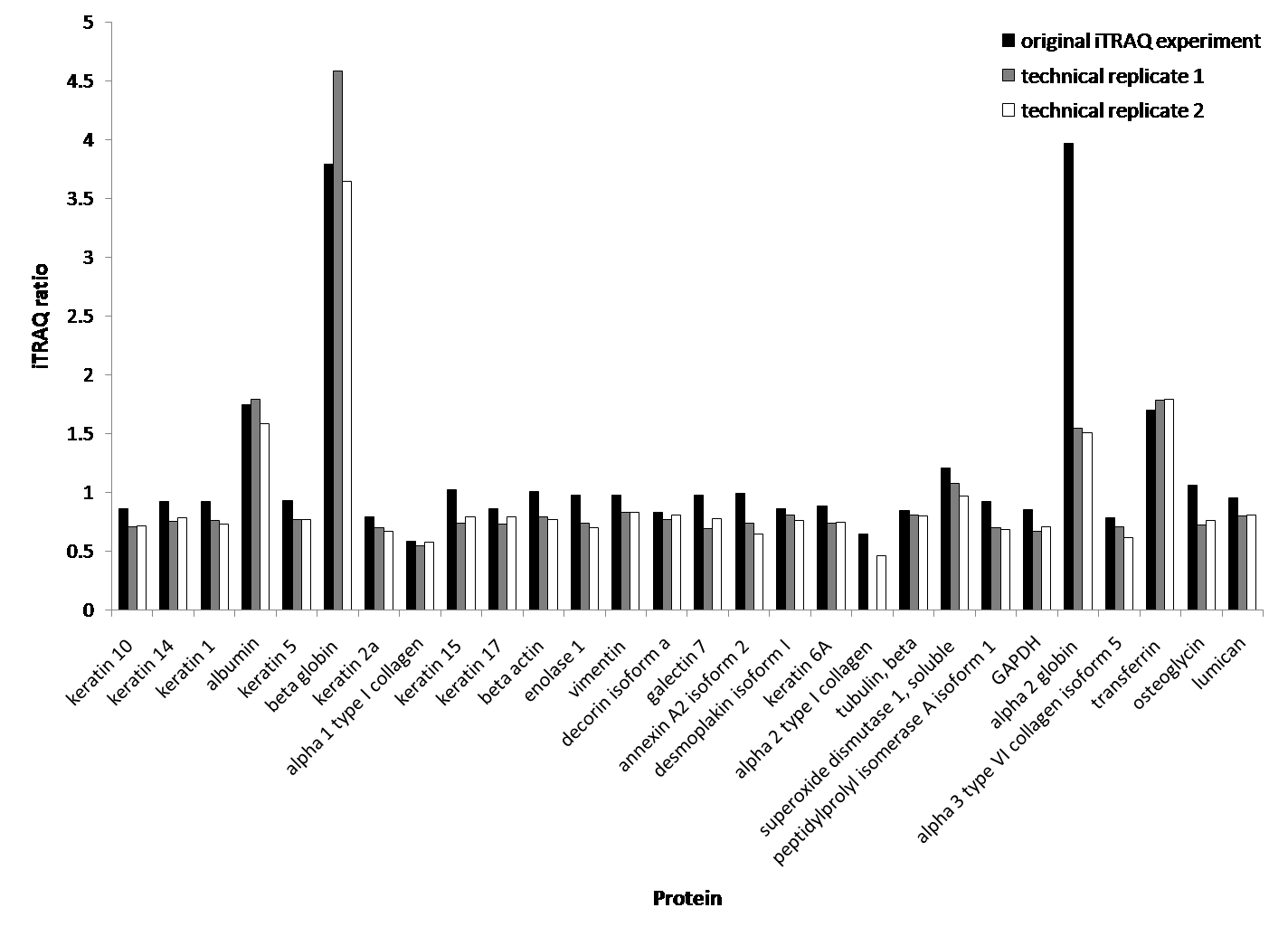

Supplement: File S2 — Figures S1, S2 and S3. Figure S1 SDS PAGE of skin tissue lysates prepared in different buffers. Figure S2 Western Blot analysis of control and test biopsy samples from 2 volunteers showing expression of caspase-14. Figure S3 Reproducibility of data in a representative experiment (F37F38) using 4- and 8-plex iTRAQ analysis. (DOCX) [file pone.0097772.s002.docx]
